# Genome-based analysis of the family *Paracoccaceae* and description of *Ostreiculturibacter nitratireducens* gen. nov., sp. nov., isolated from an oyster farm on a tidal flat

**DOI:** 10.3389/fmicb.2024.1376777

**Published:** 2024-04-30

**Authors:** Zhaobin Huang, Meiqin Li, Aharon Oren, Qiliang Lai

**Affiliations:** ^1^College of Oceanology and Food Science, Quanzhou Normal University, Quanzhou, China; ^2^Fujian Province Key Laboratory for the Development of Bioactive Material from Marine Algae, Quanzhou Normal University, Quanzhou, China; ^3^The Hebrew University of Jerusalem, Edmond J. Safra Campus, The Institute of Life Sciences, Jerusalem, Israel; ^4^Department of Marine Biology, Xiamen Ocean Vocational College, Xiamen, China; ^5^Key Laboratory of Marine Genetic Resources, Third Institute of Oceanography, Ministry of Natural Resources, Xiamen, China

**Keywords:** *Ostreiculturibacter*, polyphasic taxonomy, *Paracoccaceae*, “*Rhodobacteraceae*”, phylogenomic tree

## Abstract

Two bacterial strains, designated FR2A1^T^ and MT2-5-38, were isolated from the surface sediments of an oyster farm on a tidal flat in Quanzhou Bay, China. Both strains were Gram-stain-negative, rod-shaped, aerobic, catalase-positive, and oxidase-positive. The 16S rRNA gene sequences of the two strains were 100% identical and had the highest similarity (97.1%) with *Phaeovulum vinaykumarii* JA123^T^. The average nucleotide identity (ANI) value and digital DNA–DNA hybridization (DDH) value indicated that the two strains belonged to a single species. Gene annotation revealed that the two strains contained a gene cluster for nitrate reduction and a gene cluster for sulfur oxidation, indicating a possible role in N and S cycling in the tidal flat sediment. The phylogeny inferred from the 16S rRNA gene and 120 conserved proteins indicated that the two strains formed a distinct monophyletic clade within the family *Paracoccaceae*. The respiratory quinone was Q-10. The major fatty acids consisted of summed feature 8 (C_18:1_*ω*7*c* and/or C_18:1_*ω*6*c*) and C_18:0_. The polar lipids consisted of phosphatidylethanolamine, phosphatidylglycerol, and several unidentified phospholipids. Based on the above characteristics, strains FR2A1^T^ and MT2-5-38 represent a novel genus and a novel species, for which we propose the name *Ostreiculturibacter nitratireducens* gen. nov., sp. nov. The type strain is FR2A1^T^ (=MCCC 1K08809^T^ = KCTC 8317^T^). Phylogenomic analysis of 1,606 high-quality genomes of the family *Paracoccaceae*, including type strains, non-type strains, and uncultivated bacteria, was performed using the Genome Taxonomic Database Toolkit (GTDB-Tk), and the average amino acid identity (AAI) value of the phylogenetic clade was estimated. We found that 35 species of the family *Paracoccaceae* needed re-classification, and an AAI value of 70% was chosen as the genus boundary within the family *Paracoccaceae*.

## Introduction

The family *Paracoccaceae* (illegitimate synonym: “*Rhodobacteraceae*”) comprises the majority of the *Alphaproteobacteria* in marine habitats and displays a large phenotypic, genotypic, and metabolic diversity (Simon et al., 2017). In previous studies, the assignment of isolates (or genomes) to a species and/or genus of the family *Paracoccaceae* mainly depended on 16S rRNA gene phylogeny, which often resulted in misclassifications (Liang et al., 2021). To give a better resolution of the taxonomy of the family *Paracoccaceae*, phylogenomic analysis based on bacterial ubiquitous gene sets should be performed (Hördt et al., 2020; Liang et al., 2021; Zhang et al., 2023). Thus, the family *Roseobacteraceae* was split off from the representatives of the family *Rhodobacteraceae* in 2021, based on core-genome phylogeny (Liang et al., 2021).

In 2022, the name *Paracoccaceae* was proposed to replace the name *Rhodobacteraceae*, which is illegitimate because it contravenes Rule 51 of the International Code of Nomenclature of Prokaryotes (Göker, 2022). At the time of writing, 92 genera were included in the family *Paracoccaceae*.^1^ Bacterial species delineation based on genomic metrics is generally accepted, such as average nucleotide identity (ANI) and digital DNA–DNA hybridization (DDH) estimates, whereas a consensus genomic metric boundary for a genus delineation of the family *Paracoccaceae* is still lacking. In addition, misclassifications have occurred when using 16S rRNA gene phylogeny or including a part of type strains in the phylogenomic reconstruction. Thus, a comprehensive phylogenomic analysis of the family *Paracoccaceae* is necessary, using large datasets.

In this study, two strains, designated MT2-5-38 and FR2A1^T^, were isolated from the surface sediments of an oyster farm on a tidal flat in Quanzhou Bay, Fujian Province, China, in 2019 and 2023, respectively. The 16S rRNA gene sequence of the two strains was found to be 100% identical, suggesting that the organism may represent a novel species affiliated to the family *Paracoccaceae*. This study aimed to determine the taxonomic position of the strains. In addition, a comprehensive phylogenomic analysis of the family *Paracoccaceae* was performed based on the available genomes, including type strains, non-type strains, and uncultivated bacteria. The combination of phylogenomic analysis and average amino acid identity (AAI) metrics was used to elucidate the taxonomy of the family *Paracoccaceae*.

## Materials and methods

### Strain isolation and cultivation

Two bacterial strains designated MT2-5-38 and FR2A1^T^ were isolated from surface sediments of an oyster farm on a tidal flat in Quanzhou Bay (24°86′ N, 118°68′ E), Fujian Province, China, in March 2019 (Huang et al., 2021) and in August 2023, respectively. For the isolation of strain FR2A1^T^, 1 g surface sediment was diluted in 9 mL sterile seawater, and samples of 10× serial dilutions were spread onto marine R2A agar culture medium (R2A powder dissolved with natural seawater, adding 1.5% agar [BD]) and incubated at 28°C for 14 days. Strain FR2A1^T^ was picked and streaked onto MA (Marine Broth 2216 [BD] plus 1.5% Agar [BD]). Strain MT2-5-38 was isolated using a similar protocol (Huang et al., 2021). Briefly, 0.1 g surface sediment was diluted in 0.9 mL sterile seawater, and dilutions were spread onto MA plates.

The strains were stored at −80°C with 20% glycerol (v/v) and deposited in the Marine Culture Collection of China (MCCC) and the Korean Collection for Type Cultures (KCTC).

### Phylogeny of the 16S rRNA gene

The genomic DNA of strain FR2A1^T^ was extracted from fresh cells using the Bacterial Genomic Extraction Kit (SaiBaisheng, Co., Ltd., Shanghai, China). The 16S rRNA gene was amplified using bacterial primers Eubac27F and 1492R (Delong, 1992) with *Ex* Taq (TaKaRa) in 50 μL PCR system. Then, the PCR product was ligated into the pMD19-T vector (TaKaRa) and chemically transformed into competent *Escherichia coli* DH5α cells. A positive clone was selected and used for Sanger sequencing with the vector primer. The nearly complete 16S rRNA gene sequence of strain FR2A1^T^ was assembled using DNAMAN version 8. The partial 16S rRNA gene sequence of strain MT2-5-38 was determined in our previous study and deposited in GenBank under accession number MT829653 (Huang et al., 2021).

The close relatives of strain FR2A1^T^ and strain MT2-5-38 were searched, and their 16S rRNA gene sequences were downloaded from the EzBioCloud database (Yoon et al., 2017a) and the NCBI nucleotide database.^2^ Then, the sequences were subjected to multiple alignments by the Clustal W program implemented in MEGA 7.0 (Kumar et al., 2016). The phylogenetic tree was constructed based on two algorithms, neighbor-joining and maximum-likelihood, with 1,000 bootstraps using MEGA 7.0. The models used in neighbor-joining tree and maximum-likelihood tree were maximum composite likelihood (MCL) and K2 + G + I, respectively.

### BOX-PCR fingerprinting

BOX-PCR fingerprinting of strains FR2A1^T^ and MT2-5-38 was carried out following the protocol of Lanoot et al. (2004). Briefly, the BOX-PCR reaction was performed using 5 μL 10× *Ex* buffer, 2 μL 10 μM BOXA1R primer, 4 μL dNTP (2.5 mM), 0.25 μL *Ex* Taq (5 U/μL, TaKaRa), and 50 ng DNA. The PCR program consisted of 95°C for 7 min, 30 cycles of 90°C for 30 s, 53°C for 1 min, 65°C for 8 min, and 65°C for 16 min. Finally, PCR products were separated and visualized by using 2% agarose electrophoresis.

### Whole genome sequencing and genome annotation

The whole genome sequences of strain FR2A1^T^ and strain MT2-5-38 were determined using the Illumina NovaSeq platform (Shanghai Majorbio Bio-Pharm Technology Co., Ltd., Shanghai, China). The raw paired-end reads were trimmed using sickle^3^ with a length of 50 bp (−l 50) and quality score of 20 (−q 20). The clean reads were then assembled into contigs using SPAdes v3.8.0 (Bankevich et al., 2012). Contigs shorter than 1 kb were removed from the assembly. The complete 16S rRNA gene sequence was extracted from the whole genome sequences using RNAmmer (Lagesen et al., 2007). Genome quality (completeness and contamination) and classification were evaluated using CheckM v1.2.0 (Parks et al., 2015).

Gene prediction was performed using GeneMarkS (Besemer et al., 2001), and gene annotation was carried out using the RAST server (Aziz et al., 2008) and KAAS system.^4^ Functional proteins with the best similarities to close relatives were searched using the BLASTp program against the *nr* database with *e*-value cutoff of 1e−5 (Camacho et al., 2009).

### Genomic relatedness and phylogenomic tree

Digital DNA–DNA Hybridization (DDH, Formula 2 as recommended), average nucleotide identity (ANI), and amino acid identity (AAI) values were estimated using the GGDC website,^5^ ANI Calculator (Yoon et al., 2017b), and CompareM,^6^ respectively.

The genomes affiliated to the family *Paracoccaceae* were searched and downloaded from the genome portal of NCBI^7^ as of 25 August 2023. A total of 4,316 genomes, including type strains, non-type strains, and uncultivated bacteria, were obtained and used for genome quality estimation. The genome quality was estimated using CheckM v.1.2.0 (Parks et al., 2015). Genomes with <90% completeness and >5% contamination were excluded from the following study. The phylogenomic tree was constructed using the GTDB-Tk 1.3.0 based on 120 ubiquitously conserved bacterial proteins (Chaumeil et al., 2019). The tree was visualized using the Interactive Tree of Life (iTOL) online (Letunic and Bork, 2007).

### Phenotypic characterization

Strains FR2A1^T^ and MT2-5-38, together with the reference strain *Phaeovulum vinaykumarii* JA123^T^ (=DSM 18714^T^), were maintained under identical conditions for phenotypic comparison. Colony morphology was recorded on MA after incubation at 30°C for 2 days. Gram staining was carried out using a Gram staining kit (Hangzhou Microbial Reagent, Co. Ltd.). Catalase activity was tested using a 3% H_2_O_2_ solution. Oxidase activity was tested using the oxidase reagent (bioMérieux, France). The growth temperature range was determined under various temperatures (4, 10, 15, 20, 25, 28, 30, 35, 40, and 45°C) for 1 week. Anaerobic culture was tested in 10 mL MB in 50 mL anaerobic flasks according to our previously documented method (Liu et al., 2019). Physiological and biochemical characterization was carried out at 30°C using API ZYM, API 20NE, and API 20E strips according to the manufacturer’s instructions (bioMérieux, France).

### Chemotaxonomic characterization

The respiratory quinone of strain FR2A1^T^ was extracted as described previously (Komagata and Suzuki, 1987) and assayed by using reversed-phase high-performance liquid chromatography (Agilent 1200). For cellular fatty acid analyses, strains FR2A1^T^, MT2-5-38, and *P. vinaykumarii* JA123^T^ were cultured in MB at 30°C with shaking at 160 rpm for 2 days. The biomass was collected using centrifugation at 6,000 rpm for 10 min. The cellular fatty acids were saponified, methylated, extracted, and identified following the standard MIDI protocol (Sherlock Microbial Identification System, version 6B). For polar lipid analysis, strain FR2A1^T^ was cultured in 100 mL MB medium for 2 days, and cells were harvested by centrifugation at 6,000 rpm. Polar lipids were extracted using a chloroform/methanol system and analyzed using one- and two-dimensional TLC using Merck silica gel 60 F254 aluminum-backed thin-layer plates. Phospholipids were detected by spraying the plate with molybdenum blue.

### Nucleotide sequences

The GenBank/EMBL/DDBJ accession numbers of the 16S rRNA gene sequence of strains FR2A1^T^ and MT2-5-38 are OR533672 and MT829653, respectively. The whole genome sequences of strain FR2A1^T^ and strain MT2-5-38 have been deposited at GenBank under the accession numbers JAVQHL000000000 and JAVQHM000000000, respectively.

## Results and discussion

### 16S rRNA gene sequence phylogeny

**Figure 1:**
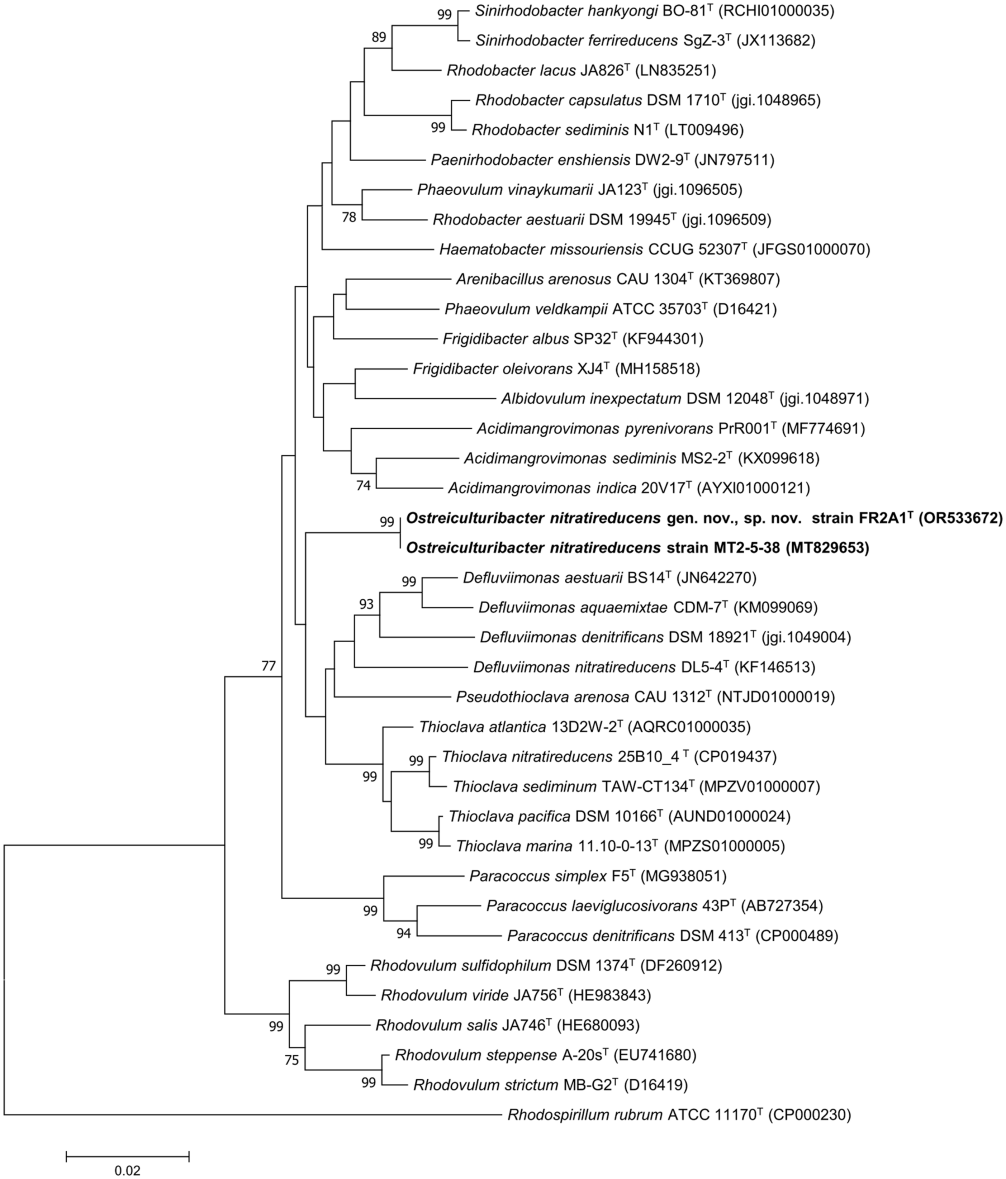
Phylogenetic analysis based on 16S rRNA gene sequences. The tree was constructed using the neighbor-joining method. *Rhodospirillum rubrum* ATCC 11170^T^ (CP000230) was selected as the outgroup. The new isolates are marked in bold. Bootstrapping was carried out with 1,000 replicates. Branch node values below 70% are not shown. Bar, 0.02 represented the nucleotide substitution per position.

The nearly complete (1,391 bp) 16S rRNA gene sequence of strain FR2A1^T^ was obtained using Sanger sequencing. It had 100% identical sequence similarity with that of strain MT2-5-38. A sequence similarity search showed that strain FR2A1^T^ had the highest 16S rRNA gene similarity (97.1%) with *Phaeovulum vinaykumarii* JA123^T^. Phylogenetic analysis based on the 16S rRNA gene indicated that strain FR2A1^T^ and strain MT2-5-38 formed a monophyletic clade distinct from closely related genera affiliated to the family *Paracoccaceae* and may be considered a new species within a new genus (Figure 1; Supplementary Figure S1). Based on the 16S rRNA gene sequence similarity, *P. vinaykumarii* JA123^T^ (=DSM 18714^T^) was chosen as a reference strain.

### BOX-PCR fingerprinting

The BOX-PCR fingerprinting profile of strain FR2A1^T^ and strain MT2-5-38 showed distinctive electrophoresis patterns (Supplementary Figure S2), confirming that they are not clonal.

### Genomic characteristics

The whole genome sequences of strains FR2A1^T^ and MT2-5-38 were determined. The genome size of strain FR2A1^T^ was 4,009,665 bp on 12 contigs (>1 kb). The genome size of strain MT2-5-38 was 3,901,132 bp on 14 contigs (>1 kb; Table 1). The genomic G + C content of strain FR2A1^T^ and strain MT2-5-38 was 65.7 and 65.6%, respectively. The ANI value and DDH estimate between strains FR2A1^T^ and MT2-5-38 were estimated to be 99.5 and 96.7%, respectively, which strongly supports that they belong to the same species. The ANI value and DDH estimate between strain FR2A1^T^ and the closest reference strain, *P. vinaykumarii* JA123^T^, were 74.5 and 21.0%, respectively.

**Table 1:** Differential characteristics of strains FR2A1^T^ and MT2-5-38 compared to the close relative *Phaeovulum vinaykumarii* JA123^T^.

Properties	1	2	3	4^a^	5^b^
Colony color	Light white	Light white	Red		Light white
Temperature (optimum, °C)	20–45 (35–40)	20–45 (35–40)	20–40 (35)	10–40 (30–40)	15–47 (28–37)
Reduction of nitrate to nitrite	+	+	−	+	−
Hydrolysis of aesculin and gelatin	−	−	+	+	−
PNPG	w	w	−	−	−
Malic acid	−	−	+	−	−
Genome size (bp, >1 kb)	4,009,665	3,901,132	3,481,684	4,107,985	3,728,293
DNA G + C content (mol%)	65.7	65.6	68.3	65.7	63.9
Isolation source	Tidal flat sediment		Tidal water	Marine aquaculture	A marine hydrothermal area

1, strain FR2A1^T^; 2, strain MT2-5-38; 3, *Phaeovulum vinaykumarii* JA123^T^; 4, *Defluviimonas denitrificans* D9-3^T^; 5, *Thioclava pacifica* TL 2^T^.

Catalase activity and oxidase activity were positive for the tested three strains. Data were taken from ^a^Liu L. et al. (2023) and ^b^Liu et al. (2017a).

+, positive; w, weak positive; −, negative. PNPG, 4-nitrophenyl-β-d-galactopyranoside.

Functional gene prediction showed 3,919 and 3,803 genes in strain FR2A1^T^ and strain MT2-5-38, respectively. Both strains contained a full set of genes for respiratory nitrate reduction (*narI*, *narJ*, *narH*, and *narG*), responsible for the reduction of nitrate to nitrite (Supplementary Table S1), and a full gene cluster for sulfur oxidation (Sox oxidation system: *SoxABCDYZ*, Supplementary Table S1), indicating a possible role in N and S cycling in their natural environment.

### Phenotypic properties

The colonies of strains FR2A1^T^ and MT2-5-38 cultured on MA were round and 1 mm in diameter. Cells were aerobic and rod-shaped (Supplementary Figure S3). Catalase and oxidase activity was positive, similar to *P. vinaykumarii* JA123^T^. Strains FR2A1^T^ and MT2-5-38 can grow at 20–40°C, with an optimum at 35–40°C (Table 1). Strains FR2A1^T^ and MT2-5-38 were positive for esterase (C4), esterase lipase (C8), leucine arylamidase, and valine arylamidase; weakly positive for alkaline phosphatase, lipase (C14), cystine arylamidase, acid phosphatase, and naphthol-AS-BI-phosphohydrolase, α-glucosidase, and β-glucosidase; negative for trypsin, α-chymotrypsin, α-galactosidase, β-galactosidase, β-glucuronidase, N-acetyl-β-glucosaminidase, α-mannosidase, and β-fucosidase. The reduction of nitrate to nitrite is positive for strains FR2A1^T^ and MT2-5-38 but negative for *P. vinaykumarii* JA123^T^. Tryptophan deaminase is positive, similar to *P. vinaykumarii* JA123^T^. Fermentation of d-glucose was negative. The hydrolysis of arginine and urea was negative. The two strains and *P. vinaykumarii* JA123^T^ cannot use d-glucose, l-arabinose, d-mannose, d-mannitol, N-acetylglucosamine, d-maltose, potassium gluconate, capric acid, adipic acid, malic acid, trisodium citrate, and phenylacetate as sole carbon sources for growth. The two strains and *P. vinaykumarii* JA123^T^ were negative for lysine decarboxylase, ornithine decarboxylase, urease, and tryptophan deaminase. Citrate cannot be utilized. H_2_S is not produced. The Voges-Proskauer reactions were negative. No acid was produced by fermentation from glucose, mannitol, inositol, sorbitol, rhamnose, sucrose, melibiose, amygdalin, and arabinose.

### Chemotaxonomic properties

The respiratory quinone of strain FR2A1^T^ was ubiquinone 10 (Q-10), like for *P. vinaykumarii* JA123^T^ and other representatives of the family *Paracoccaceae* (Hördt et al., 2020). The predominant fatty acids (>10%) of strains FR2A1^T^ and MT2-5-38 were similar, consisting of summed feature 8 (C_18:1_ *ω*7*c* and/or C_18:1_ *ω*6*c*, SF8) and C_18:0_ (Table 2). The polar lipids consist of phosphatidylethanolamine, phosphatidylglycerol, and several unidentified phospholipids (Supplementary Figure S4).

**Table 2:** Comparison of the cellular fatty acid composition.

Fatty acid	1	2	3	4^a^	5^b^
**Saturated**					
**C**_**16:0**_	3.7	3.6	**12.2**	1.8	1.0
C_17:0_	1.7	2.2	–	tr	0.2
**C**_**18:0**_	**12.2**	**12.4**	3.9	3.9	1.7
iso-C_10:0_	–	tr	1.4	–	0.1
C_19:0_ cyclo *ω*8*c*	2.1	2.4	–	**11.5**	2.4
C_19:0_ 10-methyl	–	1.1	–	–	2.2
**Unsaturated**					
C_18:1_ *ω*7*c* 11-methyl	4.5	4.9	tr	**10.7**	0.3
C_18:1_ *ω*9*c*	–	–	1.1	tr	–
Hydroxyl					
C_10:0_ 3-OH	2.4	2.1	3.2	3.1	5.1
C_18:0_ 3-OH	4.4	2.8	tr	7.1	–
C_10:0_ 3-OH iso	–	–	1.0	–	–
**Summed features**					
SF2	1.0	1.1	1.1	tr	0.2
SF3	tr	tr	4.4	tr	0.5
**SF8**	**62.7**	**63.3**	**65.8**	**59.3**	**84.4**

1, strain FR2A1^T^; 2, strain MT2-5-38; 3, *Phaeovulum vinaykumarii* JA123^T^; 4, *D. denitrificans* D9-3^T^; 5, *T. pacifica* TL 2^T^, –, not detected; tr, trace (<1%).

Data were taken from ^a^Liu L. et al. (2023) and ^b^Liu et al. (2017a).^†^Summed features are groups of two or three fatty acids that cannot be separated by GLC using the MIDI system. Summed feature 2 comprised C_12:0_ aldehyde and unknown 10.9283, summed feature 3 comprised C_16:1_ω7c and C_16:1_ω6c, and summed feature 8 comprised C_18:1_ ω7c and/or C_18:1_ ω6c. The major fatty acids (>10%) are maked bold.

### Phylogenomic analysis of the family *Paracoccaceae*

**Figure 2:**
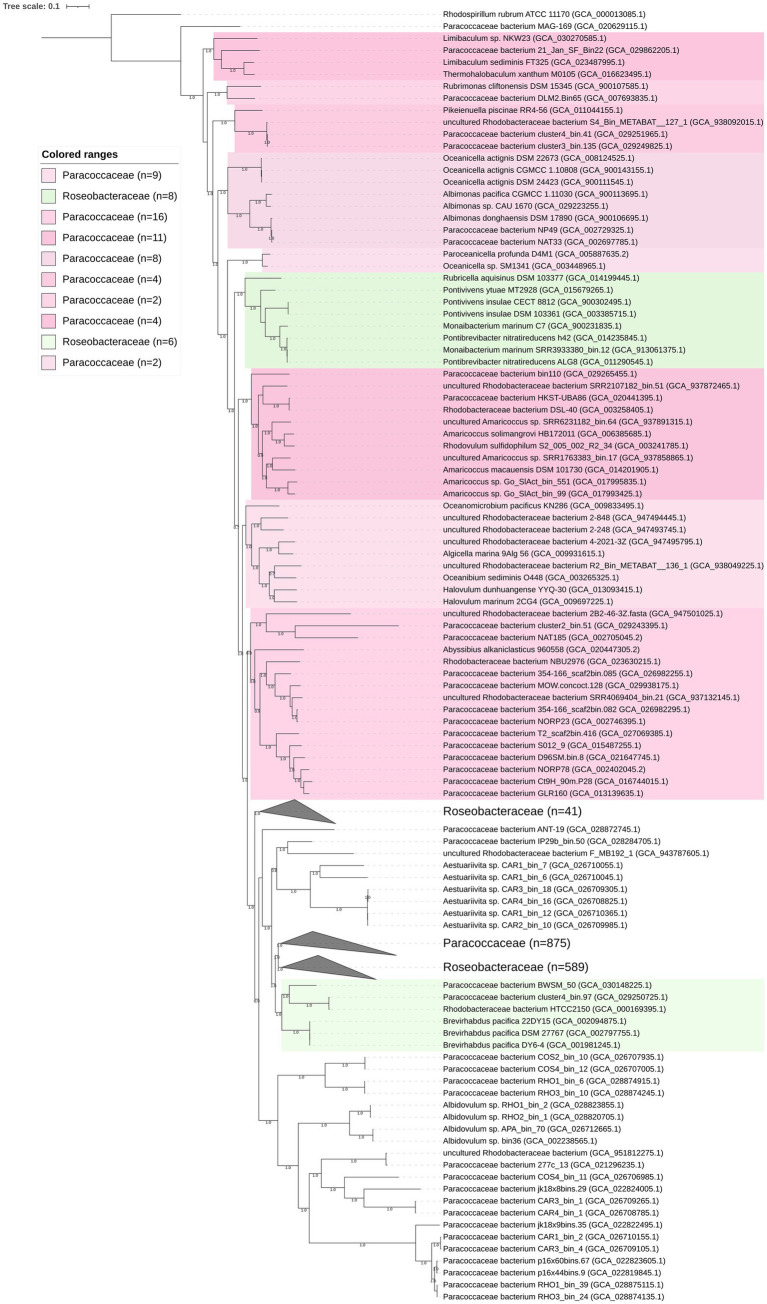
Phylogenomic tree of the family *Paracoccaceae* constructed based on bacterial 120 conserved proteins. The bootstrap values on the nodes are displayed by >70. Bar 0.1 represents the amino acid substitution per position. Based on the current taxonomic system in LPSN, *Paracoccaceae* and *Roseobacteraceae* are marked as light pink and light green, respectively. The numbers in the brackets are the genomes in the clade.

A phylogenomic tree including 1,606 high-quality genomes was constructed based on 120 conserved bacterial proteins using GTDB-Tk 1.3.0, and the AAI values of the phylogenetic clades were estimated. The studied genomes included not only the representatives of the family *Paracoccaceae* but also the representatives of the family *Roseobacteraceae*, indicating that taxonomic correction was needed. The family *Roseobacteraceae* was proposed in 2021 based on core-genome phylogeny; it was split off from the family *Paracoccaceae* (Liang et al., 2021). The genomic characteristics (genome size, G + C content, and AAI values) are listed in Supplementary Table S2. The two families formed multiple clades (Figure 2; Supplementary Figures S5, S6). Our study did not resolve the two families. Whether the family *Roseobacteraceae* needs to be re-merged into the *Paracoccaceae* needs further investigation.

The phylogenomic tree constructed based on the 120 ubiquitously conserved bacterial proteins showed that strain FR2A1^T^ formed a distinct monophyletic branch with an uncultivated bacterium bin.37 (GCA_024742695.1), a bacterium found in the phycosphere of a toxic marine dinoflagellate (*Alexandrium tamarense*), which was separated from other genera affiliated to the family *Paracoccaceae* (Supplementary Figure S5). The genome size of the bacterium bin.37 was 4.2 Mb with a genomic G + C content of 69.2%. Gene prediction showed 4,125 functional genes in the bacterium bin.37. A gene cluster for sulfur oxidation (*SoxABCDYZ*) was also annotated, but the gene cluster for nitrate reduction was not found (Supplementary Table S1). The ANI and AAI values between strain FR2A1^T^ and bacterium bin.37 were 75.9 and 71.8%, respectively. Thus, our phylogenomic analysis and genomic relatedness strongly supported that the bacterium bin.37 belonged to the same genus as strain FR2A1^T^.

In the course of the phylogenomic analysis of the 1,606 genomes of the family *Paracoccaceae*, we found discrepancies in the taxonomic positions of a few members, as elucidated below.

### *Thermohalobaculum* and *Limibaculum*

The genus *Thermohalobaculum* with type species *Thermohalobaculum xanthum* was proposed in 2021 (Pan et al., 2021). *T. xanthum* M0105^T^ and *Limibaculum sediminis* FT325^T^ are grouped together in the phylogenomic tree (Figure 2). The AAI and ANI values between *T. xanthum* M0105^T^ and *L. sediminis* FT325^T^ were 81.7 and 82.0%, respectively. The phylogeny of the 16S rRNA gene sequences placed the two strains into a highly supported clade (bootstrap of 99%), which is neighbored by *Limibaculum halophilum* (type species of *Limibaculum*) and *Rubrimonas* representatives (Supplementary Figure S7). The analysis supported that *T. xanthum* and *L. sediminis* could be merged into a single genus. Thus, we propose the transfer of *L. sediminis* to the genus *Thermohalobaculum* as *Thermohalobaculum sediminis* comb. nov. The genome size of *Thermohalobaculum* was 4.1–4.3 Mbp. The genomic G + C content of *Thermohalobaculum* was 67.9–69.6%.

### Albimonas

The AAI values among the five *Albimonas* genomes in the tree (Figure 2), including *A. pacifica* CGMCC 1.11030^T^ and *A. donghaensis* DSM 17890^T^, were 72.1–99.8%. The genome size of *Albimonas* was 5.0–6.0 Mbp. The genomic G + C content was 70.9–72.9% (Supplementary Table S2).

### *Pontivivens*, *Monaibacterium*, and *Pontibrevibacter*

*Pontivivens* currently contains two species, *P. insulae* (type species) and *P. ytuae* (Parte et al., 2020). However, the two species did not form a node in the phylogenomic tree (Figure 2). *Monaibacterium marinum* C7^T^ and *Pontibrevibacter nitratireducens* h42^T^ formed a highly supported clade, sharing AAI and ANI values of 81.5 and 78.2%, respectively, showing that they could be considered representatives of the same genus. The four species, *P. insulae*, *P. ytuae*, *M. marinum*, and *Pontibrevibacter nitratireducens*, were tightly clustered with 100% bootstrap values, sharing AAI values of 70.0–100%. The AAI values between *Rubricella aquisinus* DSM 103377^T^ and the eight close relatives were 65.3–66.5%. Thus, it is reasonable to merge the four species *P. insulae*, *P. ytuae*, *M. marinum*, and *Pontibrevibacter nitratireducens* into a single genus, separated from the genus *Rubricella*. Based on priority, *M. marinum* and *P. nitratireducens* should be merged into the genus *Pontivivens* as *Pontivivens marinum* comb. nov. and *Pontivivens nitratireducens* comb. nov., respectively. The genome size of *Pontivivens* representatives was 3.1–4.2 Mbp. The genomic G + C content was 58.9–67.2% (Supplementary Table S2).

### *Amylibacter* and *Neptunicoccus*

**Figure 3:**
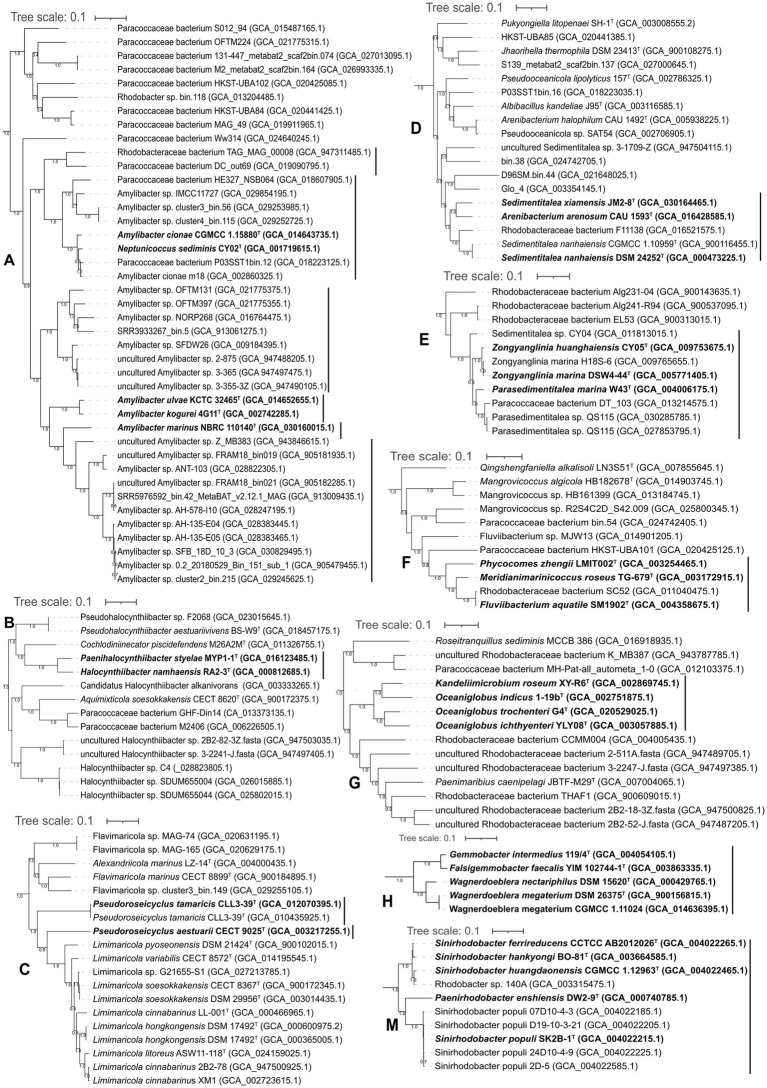

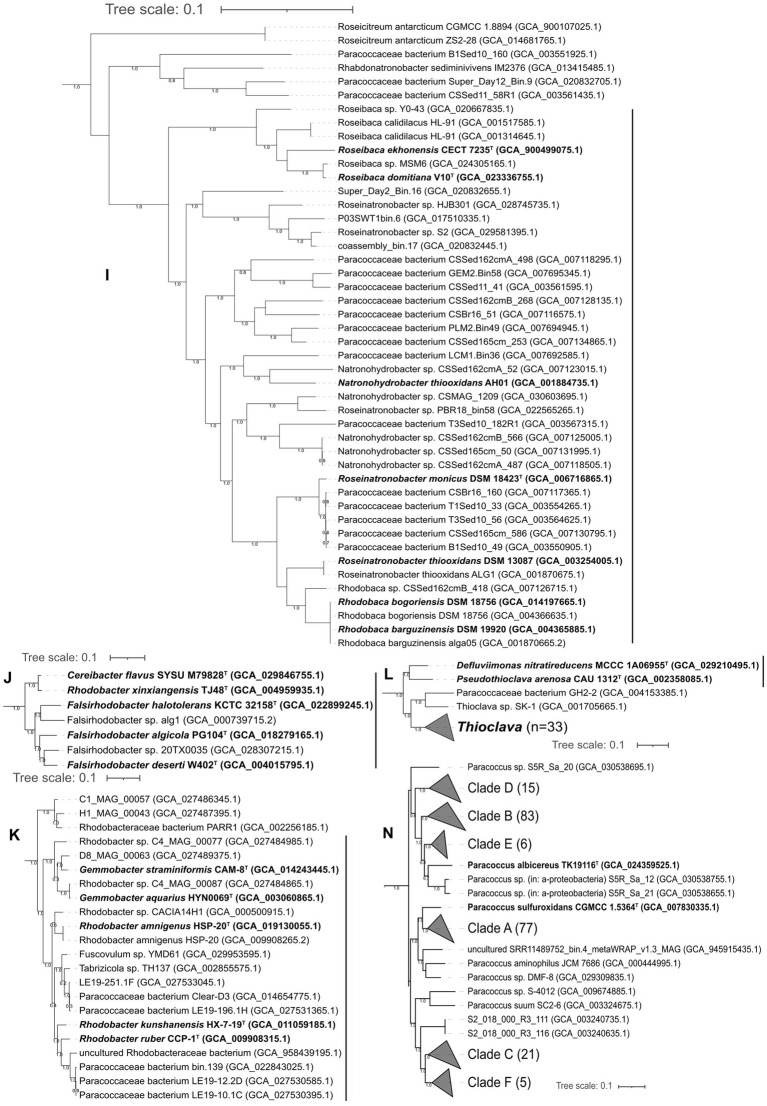
Phylogenomic tree of the genomes with discrepancies. (A-N) different clades. The species with reclassification are marked in bold.

**Figure 4:**
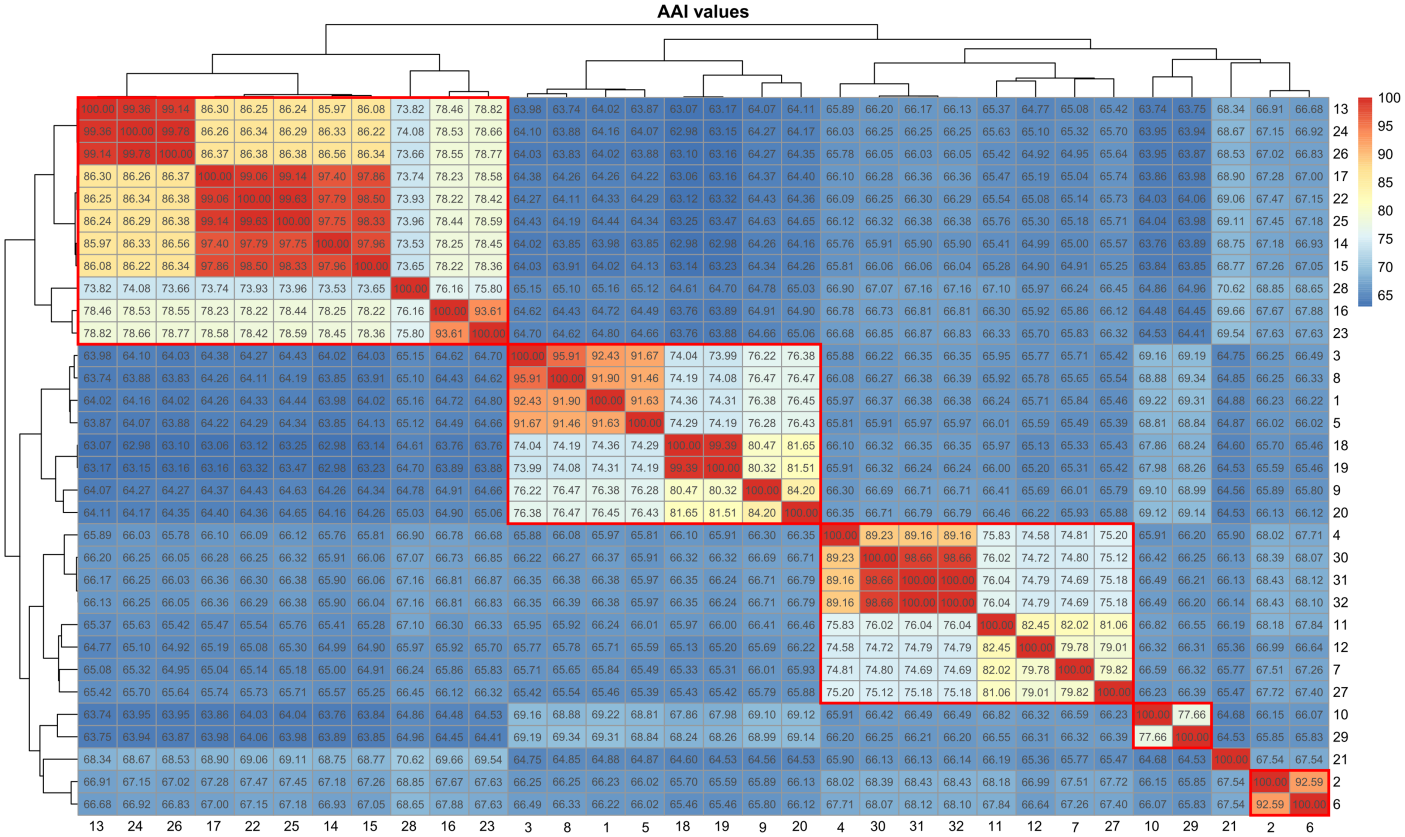
Heatmap showing the AAI values among the representatives of *Amylibacter* and *Neptunicoccus*. 1, *Neptunicoccus sediminis* CY02^T^; 2, *Amylibacter kogurei* 4G11^T^; 3, *Amylibacter cionae* m18 (GCA_002860325.1); 4, *Amylibacter* sp. SFDW26 (GCA_009184395.1); 5, *Amylibacter cionae* CGMCC 1.15880^T^; 6, *Amylibacter ulvae* KCTC 32465^T^; 7, *Amylibacter* sp. NORP268 (GCA_016764475.1); 8, *Paracoccaceae* bacterium P03SST1bin.12 (GCA_018223125.1); 9, *Paracoccaceae* bacterium HE327_NSB064 (GCA_018607905.1); 10, *Paracoccaceae* bacterium DC_out69 (GCA_019090795.1); 11, *Amylibacter* sp. OFTM397 (GCA_021775355.1); 12, *Amylibacter* sp. OFTM131 (GCA_021775375.1); 13, *Amylibacter* sp. AH-578-I10 (GCA_028247195.1); 14, *Amylibacter* sp. AH-135-E04 (GCA_028383445.1); 15, *Amylibacter* sp. AH-135-E05 (GCA_028383465.1); 16, *Amylibacter* sp. ANT-103 (GCA_028822305.1); 17, *Amylibacter* sp. cluster2_bin.215 (GCA_029245625.1); 18, *Amylibacter* sp. cluster4_bin.115 (GCA_029252725.1); 19, *Amylibacter* sp. cluster3_bin.56 (GCA_029253985.1); 20, *Amylibacter* sp. IMCC11727 (GCA_029854195.1); 21, *Amylibacter marinus* NBRC 110140^T^; 22, *Amylibacter* sp. SFB_18D_10_3 (GCA_030829495.1); 23, uncultured *Amylibacter* sp. FRAM18_bin019 (GCA_905181935.1); 24, uncultured *Amylibacter* sp. FRAM18_bin021 (GCA_905182285.1); 25, uncultured *Amylibacter* sp. 0.2_20180529_Bin_151_sub_1 (GCA_905479455.1); 26, SRR5976592_bin.42_MetaBAT_v2.12.1_MAG (GCA_913009435.1); 27, SRR3933267_bin.5 (GCA_913061275.1); 28, uncultured *Amylibacter* sp. Z_MB383 (GCA_943846615.1); 29, TAG_MAG_00008 (GCA_947311485.1); 30, uncultured *Amylibacter* sp. 2-875 (GCA_947488205.1); 31, uncultured *Amylibacter* sp. 3-355-3Z (GCA_947490105.1); 32, uncultured *Amylibacter* sp. 3-365 (GCA_947497475.1).

The phylogeny of *Amylibacter* genomes indicated multi-phyletic clades (Figure 3A). The heatmap of AAI values supported six distinct groups, corresponding to the six phylogenetic clades in the tree (Figure 4). First, *A. ulvae* KCTC 32465^T^ and *A. kogurei* 4G11^T^ formed a highly supported clade, sharing an AAI value of 92.6%, which was distantly separated from the type species *A. marinus*. Thus, we propose a novel genus, *Paramylibacter* gen. nov., to accommodate the species *A. ulvae* and *A. kogurei*. The major fatty acid of *Paramylibacter* species was C_18:1_ *ω*7*c* (Nedashkovskaya et al., 2016; Wong et al., 2018). C_14:0_ was not found in *Paramylibacter*, whereas it was found in *A. marinus*. Second, *Neptunicoccus sediminis* CY02^T^ and *A. cionae* CGMCC 1.15880^T^ were closely related, indicating that they belonged to the same genus. The ANI and AAI values between *N. sediminis* CY02^T^ and *A. cionae* CGMCC 1.15880^T^ were 85.3 and 92.6%, respectively. The major polar fatty acids of *Neptunicoccus sediminis* CY02^T^ and *A. cionae* CGMCC 1.15880^T^ contained 11-methyl C_18:1_ *ω*7*c* (>10%), a fatty acid not found in *A. marinus* NBRC 110140^T^ (Wang et al., 2017; Zhang et al., 2018). Thus, *Amylibacter cionae* (Wang et al., 2017) should be transferred to the genus *Neptunicoccus* as *Neptunicoccus cionae* comb. nov. The AAI values of *Neptunicoccus* representatives were 73.8–99.4%. The genome size of *Neptunicoccus* representatives was 2.8–4.3 Mbp. The genomic G + C content was 47.5–57.5%. Third, *A. marinus* NBRC 110140^T^ formed an independent line, representing a separate genus (Figure 3A).

### *Halocynthiibacter* and *Paenihalocynthiibacter*

Phylogenetic analysis placed *Halocynthiibacter namhaensis* RA2-3^T^ and *Paenihalocynthiibacter styelae* MYP1-1^T^ into a closely related clade, sharing an AAI value of 81%, indicating that the strains belonged to the same genus (Figure 3B). *Cochlodiniinecator piscidefendens* M26A2M^T^ shared AAI values of 68.8 and 68.7% with *Halocynthiibacter namhaensis* RA2-3^T^ and *Paenihalocynthiibacter styelae* MYP1-1^T^, respectively. Based on the priority of publication, we propose the transfer of *Paenihalocynthiibacter styelae* (Kim et al., 2021) to the genus *Halocynthiibacter* as *Halocynthiibacter styelae* comb. nov. This analysis is consistent with the GTDB taxonomic system (Chaumeil et al., 2019).

### Aliiroseovarius

*Planktotalea lamellibrachiae* DSM 104669^T^ clearly clustered within the genus *Aliiroseovarius* (Supplementary Figure S6) and should therefore be classified as a species of that genus. The taxonomic position of *Planktotalea lamellibrachiae* was recently elucidated (Zhang et al., 2023). The 53 genomes of *Aliiroseovarius* shared AAI values of 72.2–100%.

### Pseudoroseicyclus

*Pseudoroseicyclus* currently includes two species with validly published names: *Pseudoroseicyclus aestuarii* (type species; Park et al., 2016) and *Pseudoroseicyclus tamaricis* (Gai et al., 2021). However, *P. aestuarii* CECT 9025^T^ and *P. tamaricis* CLL3-39^T^ formed separate branches (Figure 3C), indicating that *P. tamaricis* CLL3-39^T^ should be placed into a novel genus. This analysis is consistent with the GTDB taxonomic system (Chaumeil et al., 2019). The AAI and ANI values between *P. aestuarii* CECT 9025^T^ and *P. tamaricis* CLL3-39^T^ were 65.9 and 74.7%, respectively. In addition, phosphatidylglycerol is found in *P. tamaricis*, but it is not found in *P. aestuarii* (Gai et al., 2021). Thus, a novel genus, *Falsiroseicyclus* gen. nov., is proposed to accommodate the species *Falsiroseicyclus tamaricis* comb. nov.

### Limimaricola

*Limimaricola* representatives (11 genomes) were well grouped in the phylogenomic tree (Figure 3C). The genome size was 3.2–4.2 Mbp. The genomic G + C content was 66.7–70.3%. The AAI value of *Limimaricola* genomes was 80.6–100%.

### Marivivens

*Marivivens* representatives (11 genomes) are well grouped in the phylogenomic tree (Supplementary Figure S6). The AAI values were 71.7–100%. The genome size was 2.5–4.1 Mbp. The genomic G + C content was 54.6–60.9%.

### Yoonia

The 58 genomes affiliated to *Yoonia* were well clustered (Supplementary Figure S6), sharing AAI values of 71.4–100%.

### Cognatishimia

The AAI values calculated among the 13 genomes of *Cognatishimia* in the tree (Supplementary Figure S6) were 70.8–100%. The genome size was 3.1–4.1 Mbp. The genomic G + C content was 53.1–58.9%.

### *Sedimentitalea* and *Arenibacterium arenosum*

*Sedimentitalea* currently contains three species with validly published names: *Sedimentitalea nanhaiensis* (type species), *S. todarodis*, and *S. xiamensis*. Phylogenetic analysis based on genome sequences showed that *Sedimentitalea* and *Arenibacterium arenosum* CAU 1593^T^ formed a monophyletic clade (Figure 3D), sharing AAI values of 76.7–82.7%. We suggested that *Arenibacterium arenosum* should be moved to the genus *Sedimentitalea*, and thus *Sedimentitalea arenosa* comb. nov. is proposed. The genomic size and genomic G + C content were 4.0–4.9 Mb and 60.8–64.2%, respectively.

### *Parasedimentitalea* and *Zongyanglinia*

*Parasedimentitalea marina* W43^T^ formed a tight cluster with *Zongyanglinia huanghaiensis* CY05^T^ and *Zongyanglinia marina* DSW4-44^T^ (Figure 3E), sharing AAI values of 82.8 and 82.7%, respectively. The values could justify the classification of the three species into the same genus. This analysis is consistent with the GTDB taxonomic system (Chaumeil et al., 2019). Thus, based on priority, we proposed the transfer of *Zongyanglinia huanghaiensis* and *Zongyanglinia marina* to the genus *Parasedimentitalea* as *Parasedimentitalea huanghaiensis* comb. nov. and *Parasedimentitalea marina* comb. nov., respectively. The AAI values of the *Parasedimentitalea* genomes were 81.8–100%. The genome size of *Parasedimentitalea* was 4.4–5.6 Mb, and the genomic G + C content was 54.2–57.8%.

### Pseudophaeobacter

*Pseudophaeobacter* representatives were well grouped (Supplementary Figure S6), sharing AAI values of 76.9–99.1%.

### Tritonibacter

*Tritonibacter* representatives were well grouped (Supplementary Figure S6), sharing AAI values of 70.2–100%.

### *Meridianimarinicoccus, Phycocomes*, and *Fluviibacterium*

*Meridianimarinicoccus roseus* TG-679^T^, *Phycocomes zhengii* LMIT002^T^, and *Fluviibacterium aquatile* SM1902^T^ formed a tight cluster (Figure 3F; Supplementary Figure S5), sharing AAI values of 75.2–98.9%, respectively. We suggest grouping the three species into the same genus. This analysis is consistent with the GTDB taxonomic system (Chaumeil et al., 2019). Thus, based on priority, we propose the transfer of *Phycocomes zhengii* and *Fluviibacterium aquatile* to the genus *Meridianimarinicoccus* as *Meridianimarinicoccus zhengii* comb. nov. and *Meridianimarinicoccus aquatilis* comb. nov. The genome size was 3.9–4.6 Mbp, and the DNA G + C content was 58.2–67.0%. We also propose the classification of *Meridianimarinicoccus* in the family *Paracoccaceae*, instead of the recommended classification in the family *Roseobacteraceae* (Liang et al., 2021).

### Rhodovulum

The 43 *Rhodovulum* genomes were well grouped (Supplementary Figure S5), sharing AAI values of 72.0–100%. We propose the classification of *Rhodovulum* as a representative of the family *Paracoccaceae*, different from the proposed assignment to the family *Roseobacteraceae* (Liang et al., 2021).

### *Oceaniglobus* and *Kandeliimicrobium*

Phylogenomic analysis placed *Kandeliimicrobium roseum* XY-R6^T^ and *Oceaniglobus indicus* 1-19b^T^ into the same lineage (Figure 3G). The AAI value between *Kandeliimicrobium roseum* XY-R6^T^ and *Oceaniglobus indicus* 1-19b^T^ was 76.0%. Based on the priority, *K. roseum* should be re-classified into the genus *Oceaniglobus* as *Oceaniglobus roseus* comb. nov. The genome size was 3.7–4.6 Mbp, and the genomic G + C content was 59.0–69.0%. We also propose that *Oceaniglobus* be a representative of the family *Paracoccaceae*, different from the recommended classification in the family *Roseobacteraceae* (Liang et al., 2021).

### Pararhodobacter

The 11 *Pararhodobacter* genomes were well grouped (Supplementary Figure S5), sharing AAI values of 71.9–100%.

### *Acidimangrovimonas* and *Allgaiera*

*Acidimangrovimonas* and *Allgaiera* were tightly clustered (Supplementary Figure S5). *Acidimangrovimonas* was first proposed in 2019 with the description of *Acidimangrovimonas sediminis* (Ren et al., 2019). The authors also re-classified two species of *Defluviimonas*, *Defluviimonas indica*, and *Defluviimonas pyrenivorans*, to the genus *Acidimangrovimonas* as *Acidimangrovimonas indica* and *Acidimangrovimonas pyrenivorans* (Ren et al., 2019). A paper published in 2020 proposed the re-classification of *Defluviimonas indica* to the genus *Allgaiera* as *Allgaiera indica* (Hördt et al., 2020; Oren and Garrity, 2020a). Based on priority, the name *Acidimangrovimonas indica* has priority and should replace the name *Allgaiera indica*. The size of the *Acidimangrovimonas* genomes was 4.3–5.3 Mbp with a genomic G + C content of 66.3–67.8% (Supplementary Table S2). The AAI values of the *Acidimangrovimonas* genomes were 77.5–100%.

### Solirhodobacter

This genus currently contains one species with a validly published name, *Solirhodobacter olei* with type strain Pet-1^T^ (Chu et al., 2020). The genome size of *Solirhodobacter* (eight genomes) was 3.1–4.8 Mbp with a genomic G + C content of 63.1–69.1%. The AAI value of *Solirhodobacter* representatives was 72.4–99.8%.

### Wagnerdoeblera

The genus *Wagnerdoeblera* was proposed in 2020 to accommodate *Wagnerdoeblera nectariphila* and *Wagnerdoeblera megaterium* (Hördt et al., 2020; Oren and Garrity, 2020a). Based on the phylogenomic analysis (Figure 3H), *Falsigemmobacter faecalis* YIM 102744-1^T^ (Li et al., 2020) and *Gemmobacter intermedius* 119/4^T^ should also be included in the genus *Wagnerdoeblera*, though *Gemmobacter intermedius* was reported to be included in the effectively published genus *Falsigemmobacter* as *Falsigemmobacter intermedius* (Li et al., 2020). The genome size of *Wagnerdoeblera* genomes was 4.1–4.5 Mbp with a genomic G + C content of 62.7–66.2%. AAI values of *Wagnerdoeblera* genomes were 70.4–100%.

### *Roseibaca*, *Roseinatronobacter*, *Natronohydrobacter*, and *Rhodobaca*

**Figure 5:**
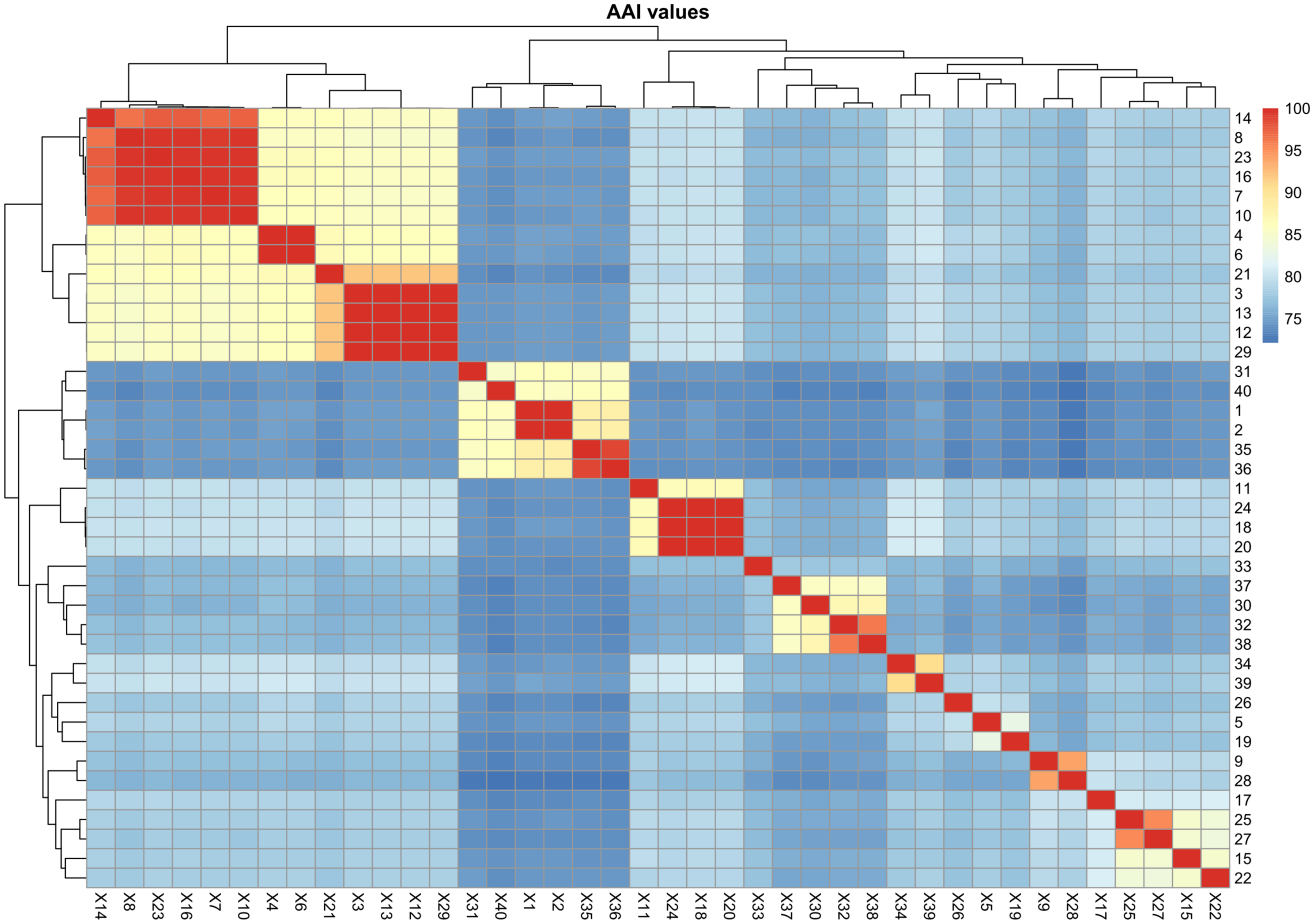
Heatmap showing the AAI values among the representatives of *Roseinatronobacter* and close relatives. 1, *Roseibaca calidilacus* HL-91 (GCA_001314645.1); 2, *Roseibaca calidilacus* HL-91 (GCA_001517585.1); 3, *Rhodobaca barguzinensis* alga05 (GCA_001870665.2); 4, *Roseinatronobacter thiooxidans* ALG1 (GCA_001870675.1); 5, *Natronohydrobacter thiooxidans* AH01 (GCA_001884735.1); 6, *Roseinatronobacter thiooxidans* DSM 13087 (GCA_003254005.1); 7, *Paracoccaceae* bacterium B1Sed10_49 (GCA_003550905.1); 8, *Paracoccaceae* bacterium T1Sed10_33 (GCA_003554265.1); 9, *Paracoccaceae* bacterium CSSed11_41 (GCA_003561595.1); 10, *Paracoccaceae* bacterium T3Sed10_56 (GCA_003564625.1); 11. *Paracoccaceae* bacterium T3Sed10_182R1 (GCA_003567315.1); 12, *Rhodobaca barguzinensis* DSM 19920 (GCA_004365885.1); 13, *Rhodobaca bogoriensis* DSM 18756 (GCA_004366635.1); 14, *Roseinatronobacter monicus* DSM 18423 (GCA_006716865.1); 15, *Paracoccaceae* bacterium CSBr16_51 (GCA_007116575.1); 16, *Paracoccaceae* bacterium CSBr16_160 (GCA_007117365.1); 17, *Paracoccaceae* bacterium CSSed162cmA_498 (GCA_007118295.1); 18, *Natronohydrobacter* sp. CSSed162cmA_487 (GCA_007118505.1); 19, *Natronohydrobacter* sp. CSSed162cmA_52 (GCA_007123015.1); 20, *Natronohydrobacter* sp. CSSed162cmB_566 (GCA_007125005.1); 21, *Rhodobaca* sp. CSSed162cmB_418 (GCA_007126715.1); 22, *Paracoccaceae* bacterium CSSed162cmB_268 (GCA_007128135.1); 23, *Paracoccaceae* bacterium CSSed165cm_586 (GCA_007130795.1); 24, *Natronohydrobacter* sp. CSSed165cm_50 (GCA_007131995.1); 25, *Paracoccaceae* bacterium CSSed165cm_253 (GCA_007134865.1); 26, *Paracoccaceae* bacterium LCM1.Bin36 (GCA_007692585.1); 27, *Paracoccaceae* bacterium PLM2.Bin49 (GCA_007694945.1); 28, *Paracoccaceae* bacterium GEM2.Bin58 (GCA_007695345.1); 29, *Rhodobaca bogoriensis* DSM 18756 (GCA_014197665.1); 30, *Paracoccaceae* bacterium P03SWT1bin.6 (GCA_017510335.1); 31, *Roseibaca* sp. Y0-43 (GCA_020667835.1); 32, *Paracoccaceae* bacterium coassembly_bin.17 (GCA_020832445.1); 33, *Paracoccaceae* bacterium Super_Day2_Bin.16 (GCA_020832655.1); 34, *Roseinatronobacter* sp. PBR18_bin58 (GCA_022565265.1); 35, *Roseibaca domitiana* V10 (GCA_023336755.1); 36, *Roseibaca* sp. MSM6 (GCA_024305165.1); 37, *Roseinatronobacter* sp. HJB301 (GCA_028745735.1); 38, *Roseinatronobacter* sp. S2 (GCA_029581395.1); 39, *Natronohydrobacter* sp. CSMAG_1209 (GCA_030603695.1); 40, *Roseibaca ekhonensis* CECT 7235 (GCA_900499075.1).

The phylogenomic tree placed four genera, *Roseibaca*, *Roseinatronobacter*, *Natronohydrobacter*, and *Rhodobaca*, in a closely related clade (Figure 3I). First, the ANI value between *Rhodobaca bogoriensis* DSM 18756^T^ and *Rhodobaca barguzinensis* alga-05^T^ was 100%, suggesting that the two strains belonged to the same species. We proposed that *Rhodobaca barguzinensis* is a later heterotypic synonym of the species *Rhodobaca bogoriensis*. Second, AAI values among the 40 genomes of the genera *Roseibaca*, *Roseinatronobacter*, *Natronohydrobacter*, and *Rhodobaca* did not give a clear boundary to discriminate between these genera (Figure 5). Thus, we suggested combining them into a single genus. Based on priority, *Roseibaca*, *Natronohydrobacter*, and *Rhodobaca* should be transferred to the genus *Roseinatronobacter*. Thus, *Roseinatronobacter ekhonensis* comb. nov., *Roseinatronobacter domitianus* comb. nov., and *Roseinatronobacter bogoriensis* comb. nov. were proposed to replace the names *Roseibaca ekhonensis*, *Roseibaca domitiana*, and *Rhodobaca bogoriensis*, respectively. The AAI values of 40 *Roseinatronobacter* genomes were 72.1–100%.

### Tabrizicola rongguiensis

*Tabrizicola rongguiensis* J26^T^ and an uncultivated bacterium (GCA_945952585.1) formed a clade within the phylogenetic tree that was distinctly separated from other *Tabrizicola* representatives (Supplementary Figure S5). The AAI value of the two genomes was 89.9% (Supplementary Table S2). Thus, *Tabrizicola rongguiensis* could be considered a novel genus, and *Aliitabrizicola rongguiensis* gen. nov., sp. nov. is proposed. The genomic size of *Aliitabrizicola* is 3.9–4.2 Mbp with a genomic G + C content of 64.2–65.2%.

### Falsirhodobacter

*Falsirhodobacter halotolerans* KCTC 32158^T^, together with *Cereibacter flavus* SYSU M79828^T^ and *Rhodobacter xinxiangensis* TJ48^T^, were closely related in the phylogenetic tree (Figure 3J). *Cereibacter flavus* SYSU M79828^T^ and *Rhodobacter xinxiangensis* TJ48^T^ could be placed into the genus *Falsirhodobacter* as *Falsirhodobacter flavus* comb. nov. and *Falsirhodobacter xinxiangensis* comb. nov., respectively. This analysis is consistent with the GTDB taxonomic system (Chaumeil et al., 2019). The genomic size of *Falsirhodobacter* is 2.8–4.0 Mbp with a genomic G + C content of 60.3–66.7%. The AAI value of the *Falsirhodobacter* genomes was 71.5–92.6%.

### Gemmobacter

The phylogeny of *Gemmobacter* genomes indicated multi-phyletic clades (Figure 3K; Supplementary Figure S5). First, *Gemmobacter aquatilis* DSM 3857^T^ and another 15 genomes formed a tight clade, sharing AAI values of 75.7–100%. The ANI and AAI between *G. nanjingensis* KCTC 23298^T^ and *G. caeni* CGMCC 1.7745^T^ were 98.3 and 99%, respectively, indicating they represented a single species. Based on priority, *G. nanjingensis* is a later heterotypic synonym of *G. caeni*. Second, *Gemmobacter aestuarii* CC-PW-75^T^ and an uncultivated bacterium ACE_PRO37 (GCA_019454225.1) formed a clade in the phylogenetic tree, which was distinctly separated from other *Gemmobacter* representatives. The AAI value of *Gemmobacter aestuarii* CC-PW-75^T^ and the uncultivated bacterium ACE_PRO37 was 71.6%. Thus, *Gemmobacter aestuarii* could be considered a representative of a novel genus, for which *Aliigemmobacter aestuarii* gen. nov., sp. nov. is proposed. The genomic size of *Aliigemmobacter* is 3.9–4.2 Mbp with a DNA G + C content of 64.2–65.2%. Third, *Gemmobacter tilapiae* KCTC 23310^T^ formed an independent monophyletic line, which represented a novel genus. Thus, *Neogemmobacter* gen. nov. is proposed. The type species is *Neogemmobacter tilapiae* comb. nov. Finally, *Rhodobacter ruber* CCP-1^T^*, Rhodobacter kunshanensis* HX-7-19^T^, *Rhodobacter amnigenus* HSP-20^T^, *Gemmobacter aquarius* HYN0069^T^, and *Gemmobacter straminiformis* CAM-8^T^ formed a tight cluster (Figure 3K), sharing AAI values of 74.6–100%. Thus, these five species could be moved to a novel genus, and *Paragemmobacter* gen. nov. is proposed. The genomic size was 3.6–4.7 Mbp. The genomic G + C content was 61.7–66.6%. The type species is *Paragemmobacter straminiformis* comb. nov.

### Pseudotabrizicola

*Pseudotabrizicola* representatives were clustered well (Supplementary Figure S5), sharing AAI values of 75.0–100%. The genomic size was 2.9–5.0 Mbp.

### Stagnihabitans

The nine genomes affiliated to the genus *Stagnihabitans* clustered together (Supplementary Figure S5), sharing AAI values of 70.4–99.1%. The genome size was 2.7–4.9 Mb. The genomic G + C content was 59.1–66.0%.

### Cypionkella

The 18 genomes affiliated to the genus *Cypionkella* clustered together (Supplementary Figure S5), sharing AAI values of 69.9–99.9%. The genome size was 2.7–5.1 Mb. The genomic G + C content was 57.7–62.4%.

### Tabrizicola

*Tabrizicola* representatives and an isolate named *Rhodobacter calidifons* M3P7 clustered well in the phylogenetic tree (Supplementary Figure S3), sharing AAI values of 75.4–100%.

### Defluviimonas

The *Defluviimonas* representatives clustered well in the phylogenetic tree (Supplementary Figure S5). The AAI values among the *Defluviimonas* representatives were 70.6–100%.

### Thioclava

The *Thioclava* representatives clustered well in the phylogenetic tree (Supplementary Figure S5). The AAI values among the *Thioclava* representatives were 77.3–100%.

### Frigidibacter

The *Frigidibacter* representatives clustered well in the phylogenetic tree (Supplementary Figure S5). The AAI values among the *Frigidibacter* representatives were 74.2–100%.

### *Pseudothioclava* and *Defluviimonas nitratireducens*

*Pseudothioclava* currently contains a single species with a validly published name, *Pseudothioclava arenosa* (Kim et al., 2019; Oren and Garrity, 2020a). *Pseudothioclava arenosa* CAU 1312^T^ and *Defluviimonas nitratireducens* MCCC 1A06955^T^ clustered together (Figure 3L), sharing AAI and ANI values of 78.3 and 78.6%, respectively. Thus, we suggest that *Defluviimonas nitratireducens* should be re-classified into the genus *Pseudothioclava* as *Pseudothioclava nitratireducens* comb. nov.

### Sedimentimonas

*Sedimentimonas* currently includes a single species, *Sedimentimonas flavescens* (Mu et al., 2022). The *Sedimentimonas* representatives formed a monophyletic clade (Supplementary Figure S5), sharing AAI values of 79.1–98.9%. The genome size of *Sedimentimonas* was 3.1–3.8 Mbp. The DNA G + C content was 64.1–67.0%.

### *Sinirhodobacter* and *Paenirhodobacter*

*Paenirhodobacter* was proposed and validly published in 2014 with the type species *Paenirhodobacter enshiensis* (Wang et al., 2014). *Sinirhodobacter* (former name *Sinorhodobacter*) was proposed in 2013 with the type species *Sinirhodobacter ferrireducens* (Yang et al., 2013), but the name was validly published in 2018 (Oren and Garrity, 2018). *Paenirhodobacter enshiensis* DW2-9^T^ and the *Sinirhodobacter* representatives formed a monophyletic clade (Figure 3M), sharing AAI values of 73.3–98.4%. These data supported that *Paenirhodobacter* and *Sinirhodobacter* could be merged into a single genus. Thus, based on priority, the *Sinirhodobacter* representatives should be transferred to the genus *Paenirhodobacter*. *Sinirhodobacter ferrireducens*, *Sinirhodobacter hungdaonensis*, *Sinirhodobacter populi*, and *Sinirhodobacter hankyongi* should be renamed as *Paenirhodobacter ferrireducens* comb. nov., *Paenirhodobacter hungdaonensis* comb. nov., *Paenirhodobacter populi* comb. nov., and *Paenirhodobacter hankyongi* comb. nov., respectively. The result is consistent with the taxonomic system in GTDB (Chaumeil et al., 2019). The genome size of *Paenirhodobacter* was 3.4–4.8 Mbp. The genomic G + C content was 65.4–68.3%.

### Rhodobacter

The type species of *Rhodobacter* is *Rhodobacter capsulatus* with type strain DSM 1710^T^ (Imhoff et al., 1984). *R. capsulatus* DSM 1710^T^, *R. viridis* JA737^T^, *R. aestuarii* JA296^T^, *R. maris* JA276^T^, and 17 additional genomes formed a tight clade (Supplementary Figure S5), sharing AAI values of 79.1–100%. The genome size of *Rhodobacter* representatives was 3.6–4.2 Mbp. The genomic G + C content was 61.1–66.7%.

### Phaeovulum

The genus *Phaeovulum* currently contains two species with validly published names, *Phaeovulum veldkampii* and *Phaeovulum vinaykumarii* (Parte et al., 2020; Oren and Garrity, 2020b). However, *P. veldkampii* DSM 11550^T^ and *P. vinaykumarii* JA123^T^ did not form a cluster in the phylogenetic tree, making us question their placement. Their taxonomic position needs further study.

### Paracoccus

A total of 219 high-quality genomes affiliated to the genus *Paracoccus* were included in the phylogenomic tree (Figure 3N; Supplementary Figure S5). At the time of writing, the genus contained 81 species with validly published names,^8^ outnumbering the species of the other genera classified in the family *Paracoccaceae*. The AAI values among the 219 *Paracoccus* genomes showed at least six clades, numbered from Clade A to Clade F, to be consistent with the phylogenomic clades (Supplementary Figure S8). This indicated that the genus *Paracoccus* could be split into several new genera. However, as the genus *Paraccocus* is widely used, their taxonomic position needs further study.

In summary, based on the above results of phenotypic, genomic, and chemotaxonomic characteristics, strains FR2A1^T^ and MT2-5-38 represent a novel genus and novel species within the family *Paracoccaceae*. The name *Ostreiculturibacter nitratireducens* gen. nov., sp. nov. is proposed, with type strain FR2A1^T^ (=MCCC 1K08809^T^ = KCTC 8317^T^). A second strain is MT2-5-38 (=MCCC 1 K08810). Both were isolated from the surface sediment of an oyster farm on a tidal flat in Quanzhou Bay, China. Additionally, based on the AAI values of the above phylogenomic clades, though not all clades in the family *Paracoccaceae*, the genera have a clear threshold of an AAI value of 70%. Thus, an AAI value of 70% could be considered the genus boundary within the family *Paracoccaceae*.

### Taxonomic consequences

#### Description of *Ostreiculturibacter* gen. nov.

*Ostreiculturibacter* (Os.tre.i.cul.tu.ri.bacter. L. fem. n. *ostrea*, an oyster; L. fem. n. *cultura*, cultivation; N.L. masc. n. *bacter*, a rod; N.L. masc. n. *Ostreiculturibacter*, a rod from an oyster farm).

Colonies on MB agar plates cultured for 2 days at 30°C are light-white colored, small, and round. Cells are Gram-stain-negative and rod-shaped. Catalase-positive and oxidase-positive. The quinone system is quinone Q-10. The major fatty acids are summed feature 8 (C_18:1_ *ω*7*c*/C_18:1_ *ω*6*c*) and C_18:0_. The polar lipids consisted of phosphatidylethanolamine, phosphatidylglycerol, and several unidentified phospholipids. The genome contained a gene cluster (Sox system) for sulfur oxidation. The genome size is 3.9–4.2 Mbp, calculated from the strains and a metagenome-assembled genome. The genomic G + C content is 65.6–69.2%. It was found in the tidal flat surface sediment and the phycosphere of a toxic marine dinoflagellate. The type species is *Ostreiculturibacter nitratireducens*.

#### Description of *Ostreiculturibacter nitratireducens* sp. nov.

*Ostreiculturibacter nitratireducens* (ni.tra.ti.re.du’cens. N.L. masc. n. *nitras*, nitrate; L. pres. part. *reducens*, converting to a different state; N.L. part. adj. *nitratireducens*, reducing nitrate).

The description is as given for the genus, with the following additions. Growth occurred in the temperature range of 20–45°C, with an optimum of 35–40°C. Nitrate can be reduced to nitrite. Positive for tryptophan deaminase. Weak positive for 4-nitrophenyl-β-d-galactopyranoside. Positive for alkaline phosphatase, esterase (C4), esterase lipase (C8), leucine arylamidase, valine arylamidase, acid phosphatase, and β-glucuronidase; weak positive for lipase (C14), naphthol-AS-BI-phosphohydrolase, and α-glucosidase. The hydrolysis of gelatin and aesculin is negative. Cannot use d-glucose, l-arabinose, d-mannose, d-mannitol, N-acetylglucosamine, d-maltose, potassium gluconate, capric acid, adipic acid, malic acid, trisodium citrate, and phenylacetate as sole carbon sources for growth. The genome size is 3.9–4.0 Mbp. The genomic G + C content was 65.6–65.7%. The 16S rRNA gene sequence and the whole genome sequence of strain FR2A1^T^ have been deposited at DDBJ/ENA/GenBank under the accession numbers OR533672 and JAVQHL000000000, respectively.

The type strain is FR2A1^T^ (=MCCC 1K08809^T^ = KCTC 8317^T^), and another strain is MT2-5-38 (=MCCC 1 K08810), which were isolated from the surface sediment of an oyster farm on a tidal flat in Quanzhou Bay, China.

#### Description of *Thermohalobaculum sediminis* comb. nov.

*Thermohalobaculum sediminis* (se.di’mi.nis. L. gen. n. *sediminis*, of sediment).

Basonym: *Limibaculum sediminis* Huang *et al*. 2022.

The description is as given for *Limibaculum sediminis* (Huang et al., 2022).

The type strain is FT325^T^ (=MCCC 1K07397^T^ = KCTC 92313^T^).

#### Emended description of *Pontivivens* Park *et al*. 2015

As the original authors of the genus failed to indicate the gender of the name, and the gender cannot be deduced from the names of its species published thus far, we propose the following emended etymology of the name: Pon.ti.vi’vens. L. masc. n. *pontus*, the sea; L. pres. part. *vivens*, living; N.L. neut. n. *Pontivivens*, an organism living in the sea.

#### Description of *Pontivivens marinum* comb. nov.

*Pontivivens marinum* (ma.ri’num. L. neut. n. *marinum*, marine, of the sea).

Basonym: *Monaibacterium marinum* Chernikova *et al*. 2017.

The description is as given for *Monaibacterium marinum* (Chernikova et al., 2017).

The type strain is C7^T^ (=DSM 100241^T^ = LMG 28800^T^).

#### Description of *Pontivivens nitratireducens* comb. nov.

*Pontivivens nitratireducens* (ni.tra.ti.re.du’cens. N.L. masc. n. *nitras*, nitrate; L. pres. part. *reducens*, converting to a different state; N.L. part. adj. *nitratireducens*, reducing nitrate).

Basonym: *Pontibrevibacter nitratireducens* Liang *et al*. 2022.

The description is as given for *Pontibrevibacter nitratireducens* (Liang et al., 2022).

The type strain is h42^T^ (=KCTC 72875^T^ = CGMCC 1.17849^T^ = MCCC 1K04735^T^). Another strain is ALG8 (=KCTC 82194 = MCCC 1 K04733).

#### Description of *Paramylibacter* gen. nov.

*Paramylibacter* (Par.a.my.li.bac’ter. Gr prep. *para*, beside; N.L. masc. n. *Amylibacter*, a bacterial genus; N.L. masc. n. *Paramylibacter*, beside *Amylibacter*).

Cells are Gram-stain-negative, strictly aerobic, rod-shaped, and non-motile. Catalase-positive and oxidase-positive. The major respiratory quinone is Q-10. The major fatty acid is C_18:1_ *ω*7*c*. The major polar lipids included phosphatidylglycerol, phosphatidylcholine, and an unidentified aminolipid. *Paramylibacter* was phylogenetically distinct from *Amylibacter marinus*. The genomic G + C content was ~49%. The type species is *Paramylibacter ulvae*.

#### Description of *Paramylibacter ulvae* comb. nov.

*Paramylibacter ulvae* (ul’vae. L. gen. n. *ulvae*, of *Ulva*, an algal genus).

Basonym: *Amylibacter ulvae* Nedashkovskaya *et al*. 2016.

The description is as given for *Amylibacter ulvae* (Nedashkovskaya et al., 2016).

The type strain is 6Alg 255^T^ (=KCTC 32465^T^ = KMM 6515^T^).

#### Description of *Paramylibacter kogurei* comb. nov.

*Paramylibacter kogurei* (ko.gu’re.i. N.L. gen. n. *kogurei*, of Kogure, to honor the Japanese microbiologist, Kazuhiro Kogure, in recognition of his contribution to the field of marine microbiology).

Basonym: *Amylibacter kogurei* Wong *et al*. 2018.

The description is as given for *Amylibacter kogurei* (Wong et al., 2018).

The type strain is 4G11^T^ (=KY463497^T^ = KCTC 52506^T^ = NBRC 112428^T^).

#### Description of *Neptunicoccus cionae* comb. nov.

*Neptunicoccus cionae* (ci.o’nae. N.L. gen. fem. n. *cionae*, of the sea squirt *Ciona*).

Basonym: *Amylibacter cionae* Wang *et al*. 2017.

The description is as given for *Amylibacter cionae* (Wang et al., 2017).

The type strain is H-12^T^ (=KCTC 52581^T^ = CGMCC 1.15880^T^).

#### Description of *Halocynthiibacter styelae* comb. nov.

*Halocynthiibacter styelae* (sty.e’lae. N.L. gen. fem. n. *styelae*, of *Styela*, named after the generic name of the stalked sea squirt *Styela clava*, from which the type strain was isolated).

Basonym: *Paenihalocynthiibacter styelae* Kim *et al*. 2021.

The description is as given for *Paenihalocynthiibacter styelae* (Kim et al., 2021).

The type strain is MYP1-1^T^ (=KCTC 82143^T^ = NBRC 114355^T^).

#### Description of *Falsiroseicyclus* gen. nov.

*Falsiroseicyclus* (Fal.si.ro.se.i.cy’clus. L. masc. perf. part. *falsus*, false; N.L. masc. n. *Roseicyclus*, a bacterial genus; N.L. masc. n. *Falsiroseicyclus*, a false *Roseicyclus*).

Cells are Gram-stain-negative, ovoid- or rod-shaped, and non-motile. Catalase-positive and oxidase-positive. The major respiratory quinone is Q-10. The major fatty acids (>10%) are summed feature 8 (C_18:1_ *ω*7*c*/C_18:1_ *ω*6*c*), anteiso-C_15:0_, and iso-C_15:0_. The major polar lipids included phosphatidylglycerol, phosphatidylcholine, diphosphatidylglycerol, and unidentified lipids. The genomic G + C content was ~69.6%. *Falsiroseicyclus* formed a separate phylogenetic line with *Pseudoroseicyclus.* The type species is *Falsiroseicyclus tamaricis*.

#### Description of *Falsiroseicyclus tamaricis* comb. nov.

*Falsiroseicyclus tamaricis* (ta.ma’ri.cis. L. gen. n. *tamaricis*, of the tamarix tree).

Basonym: *Pseudoroseicyclus tamaricis* Gai *et al*. 2021.

The description is given for *Pseudoroseicyclus tamaricis* (Gai et al., 2021).

The type strain is CLL3-39^T^ (=MCCC 1A14815^T^ *=* KCTC 72665^T^).

#### Description of *Sedimentitalea arenosa* comb. nov.

*Sedimentitalea arenosa* (a.re.no’sa. L. fem. adj. *arenosa*, sandy, dwelling in sand).

Basonym: *Arenibacterium arenosum* Jeong *et al*. 2024.

The description is as given for *Arenibacterium arenosum* (Jeong et al., 2022).

The type strain is CAU1593^T^ (=KCTC 82402^T^ = MCCC 1K05671^T^).

#### Description of *Parasedimentitalea huanghaiensis* comb. nov.

*Parasedimentitalea huanghaiensis* (huang.hai.en’sis. N.L. fem. adj. *huanghaiensis*, pertaining to Huanghai, the Chinese name for the Yellow Sea, the geographical origin of the type strain).

Basonym: *Zongyanglinia huanghaiensis* Xu *et al*. 2021.

The description is as given for *Zongyanglinia huanghaiensis* (Xu et al., 2021).

The type strain is CY05^T^ (=MCCC 1K04409^T^ = KCTC 62200^T^).

#### Description of *Parasedimentitalea marina* comb. nov.

*Parasedimentitalea marina* (ma.ri’na. L. fem. adj. *marina*, inhabiting the sea).

Basonym: *Pelagicola marinus* Choi *et al*. 2019.

Homotypic synonym: *Zongyanglinia marinus* (*sic*; Choi et al., 2019; Xu et al., 2021).

The description is as given for *Pelagicola marinus* (Choi et al., 2019) and *Zongyanglinia marina* (Xu et al., 2021).

The type strain is DSW4-44^T^ (=KCTC 62762^T^ = KCCM 43261^T^ = JCM 33637^T^).

#### Description of *Meridianimarinicoccus zhengii* comb. nov.

*Meridianimarinicoccus zhengii* (zheng’i.i. N.L. gen. n. *zhengii*, referring to Dr. Tianling Zheng, who contributed to studies of algicidal bacteria).

Basonym: *Phycocomes zhengii* Zhu *et al*. 2019.

The description is as given for *Phycocomes zhengii* (Zhu et al., 2019).

The type strain is LMIT002^T^ (=KCTC 62390^T^ = CICC 24357^T^).

#### Description of *Meridianimarinicoccus aquatilis* comb. nov.

*Meridianimarinicoccus aquatilis* (a.qua’ti.lis. L. fem. adj. *aquatilis*, aquatic).

Basonym: *Fluviibacterium aquatile* Sun *et al*. 2020.

The description is as given for *Fluviibacterium aquatile* (Sun et al., 2020).

The type strain is SM1902^T^ (=KCTC 72045^T^ = MCCC 1K03596^T^ = CCTCC AB 2018346^T^).

#### Description of *Oceaniglobus roseus* comb. nov.

*Oceaniglobus roseus* (ro’se.us. L. masc. n. *roseus*, pink, rose-colored, rosy).

Basonym: *Kandeliimicrobium roseum* Wang *et al*. 2018).

The description is as given for *Kandeliimicrobium roseum* (Wang et al., 2018).

The type strain is XY-R6^T^ (=MCCC 1K01498^T^ = KCTC 52266^T^ = DSM 104294^T^).

#### Description of *Roseinatronobacter ekhonensis* comb. nov.

*Roseinatronobacter ekhonensis* (ek.ho.nen’sis. N.L. masc. adj. *ekhonensis*, pertaining to Ekho Lake, the lake in Antarctica from which the organism was isolated).

Basonym: *Roseibaca ekhonensis* Labrenz *et al*. 2009.

The description is as given for *Roseibaca ekhonensis* (Labrenz et al., 2009).

The type strain is EL-50^T^ (=CECT 7235^T^ = DSM 11469^T^).

#### Description of *Roseinatronobacter domitianus* comb. nov.

*Roseinatronobacter domitianus* (do.mi.ti.a’nus. L. masc. adj. *domitianus*, belonging to the Domitian littoral).

Basonym: *Roseibaca domitiana* Labrenz *et al*. 2024.

The description is as given for *Roseibaca domitiana* (Gattoni et al., 2023).

The type strain is V10^T^ (=CECT 30319^T^ = DSM 112951^T^ = LMG 32429^T^).

#### Description of *Roseinatronobacter bogoriensis* comb. nov.

*Roseinatronobacter bogoriensis* (bo.go.ri.en’sis. N.L. masc. adj. *bogoriensis*, pertaining to Lake Bogoria, a soda lake in Kenya, Africa).

Basonym: *Rhodobaca bogoriensis* Milford *et al*. 2001.

The description is as given for *Rhodobaca bogoriensis* (Milford et al., 2000).

The type strain is LBB1^T^ (=ATCC 700920^T^ = DSM 18756^T^). Another strain is alga-05 (=DSM 19920 = VKM B-2406).

#### Description of *Wagnerdoeblera intermedia* comb. nov.

*Wagnerdoeblera intermedia* (in.ter.me’di.a. L. fem. adj. *intermedia*, in the middle, referring to the fact that the species is grouped between *Gemmobacter* and *Rhodobacter* and *Roseinatronobacter* and *Roseibaca* on the basis of 16S rRNA gene sequence similarities).

Basonym: *Gemmobacter intermedius* Kämpfer *et al*. 2015.

Homotypic synonym: *Falsigemmobacter intermedius* (Kämpfer et al. 2015) Li *et al*. 2023.

The description is as given for *Gemmobacter intermedius* (Kämpfer et al., 2015).

The type strain is 119/4^T^ (=CIP 110795^T^ = LMG 28215^T^ = CCM 8510^T^ = DSM 28642^T^).

#### Description of *Wagnerdoeblera faecalis* comb. nov.

*Wagnerdoeblera faecalis* (fae.ca’lis. N.L. fem. adj. *faecalis*, pertaining to feces, fecal).

Basonym: *Falsigemmobacter faecalis* Li *et al*. 2023.

The description is as given for *Falsigemmobacter faecalis* (Li et al., 2020).

The type strain is YIM 102744-1^T^ (=CCTCC AB 2016031^T^ = KCTC 52106^T^).

#### Description of *Aliitabrizicola* gen. nov.

*Aliitabrizicola* (A.li.i.ta.bri.zi’co.la. L. masc. adj. *alius*, other; N.L. fem. n. *Tabrizicola*, a bacterial genus; N.L. fem. n. *Aliitabrizicola*, another *Tabrizicola*).

Cells are Gram-stain-negative, rod-shaped, and non-motile. Catalase-positive and oxidase-positive. The major respiratory quinone is Q-10. The major fatty acid (>10%) is C_18:1_ *ω*7*c*. The major polar lipids include diphosphatidylglycerol, phosphatidylglycerol, phosphorylethanolamine, and unidentified lipids. *Aliitabrizicola* formed a separate phylogenetic line with *Tabrizicola.* The type species is *Allitabrizicola rongguiensis*.

#### Description of *Allitabrizicola rongguiensis* comb. nov.

*Allitabrizicola rongguiensis* (rong.gui.en’sis. N.L. fem. adj. *rongguiensis*, pertaining to the Ronggui river).

Basonym: *Tabrizicola rongguiensis* Xu *et al*. 2022.

The description is as given for *Tabrizicola rongguiensis* (Xu et al., 2022).

The type strain is J26^T^ (=GDMCC 1.2843^T^ = KCTC 92112^T^).

#### Description of *Falsirhodobacter flavus* comb. nov.

*Falsirhodobacter flavus* (fla’vus. L. masc. adj. *flavus*, yellow).

Basonym: *Cereibacter flavus* Liu *et al*. 2023.

The description is as given for *Cereibacter flavus* (Liu W. L. et al., 2023).

The type strain is SYSU M79828^T^ (=GDMCC 1.3803^T^ = KCTC 92893^T^).

#### Description of *Falsirhodobacter xinxiangensis* comb. nov.

*Falsirhodobacter xinxiangensis* (xin.xiang.en’sis. N.L. masc. adj. *xinxiangensis*, referring to Xinxiang in the Henan province, China, the area from where the type strain was isolated).

Basonym: *Rhodobacter xinxiangensis* Han *et al*. 2024.

The description is as given for *Rhodobacter xinxiangensis* (Han et al., 2020).

The type strain is TJ48^T^ (=CCTCC AB 2019120^T^ = KCTC 72510^T^).

#### Description of *Aliigemmobacter* gen. nov.

*Aliigemmobacter* (A.li.i.gem.mo.bac’ter. L. masc. adj. *alius*, other; N.L. masc. n. *Gemmobacter*, a bacterial genus; N.L. masc. n. *Aliigemmobacter*, another *Gemmobacter*).

Cells are Gram-stain-negative and rod-shaped. Catalase-positive and oxidase-positive. The major quinone is Q-10. The major fatty acids are summed feature 8 (C_18:1_ *ω*7*c* and/or C_18:1_ *ω*6*c*), C_18:0_, and C_18:1_ *ω*7*c* 11-methyl. The major polar lipids included phosphatidylethanolamine, phosphatidylmonomethylethanolamine, phosphatidylglycerol, phosphatidylcholine, and an unidentified aminolipid. *Aliigemmobacter* formed a separate phylogenetic line with *Gemmobacter.* The type species is *Aliigemmobacter aestuarii.*

#### Description of *Aliigemmobacter aestuarii* comb. nov.

*Aliigemmobacter aestuarii* (aes.tu.a’ri.i. L. gen. n. *aestuarii*, of the shallow coast, from where the type strain was isolated).

Basonym: *Gemmobacter aestuarii* Hameed *et al*. 2020.

The description is as given for *Gemmobacter aestuarii* (Hameed et al., 2020).

The type strain is CC-PW-75^T^ (=JCM 19754^T^ = BCRC 80759^T^).

#### Description of *Neogemmobacter* gen. nov.

*Neogemmobacter* (Ne.o.gem.mo.bac’ter. Gr. masc. adj. *neos*, new; N.L. masc. n. *Gemmobacter*, a bacterial genus; N.L. masc. n. *Neogemmobacter*, a new *Gemmobacter*).

Cells are Gram-stain-negative, aerobic, non-motile, and rod-shaped. Catalase-positive and oxidase-positive. Cells can produce poly-β-hydroxybutyrate. The major quinone is Q-10. The major fatty acids are C_18:1_ *ω*7*c*. The major polar lipids included phosphatidylglycerol, phosphatidylethanolamine, phosphatidylcholine, and unidentified amino lipids. *Neogemmobacter* formed a separate phylogenetic line with *Gemmobacter.* The type species is *Neogemmobacter tilapiae*.

#### Description of *Neogemmobacter tilapiae* comb. nov.

*Neogemmobacter tilapiae* (ti.la’pi.ae. L. gen. n. *tilapiae*, of *Tilapia*, the common name of tilapiine cichlid fish, referring to the isolation of the type strain from a pond for rearing Tilapia fish).

Basonym: *Gemmobacter tilapiae* Sheu et al. 2013.

The description is as given for *Gemmobacter tilapiae* (Sheu et al., 2013).

The type strain is Ruye-53^T^ (=BCRC 80261^T^ = KCTC 23310^T^).

#### Description of *Paragemmobacter* gen. nov.

*Paragemmobacter* (Pa.ra.gem.mo.bac’ter. Gr. prep. *para*, beside; N.L. masc. n. *Gemmobacter*, a bacterial genus; N.L. masc. n. *Paragemmobacter*, beside *Gemmobacter*).

Cells are Gram-stain-negative, facultatively anaerobic, non-motile, and rod-shaped. Catalase-positive and oxidase-positive. The major quinone is Q-10. The major fatty acids are summed feature 8 (C_18:1_ *ω*7*c*/C_18:1_ *ω*6*c*). The major polar lipids included phosphatidylethanolamine, phosphatidylglycerol, phosphatidylcholine, unidentified glycolipids, and unidentified amino phospholipids. *Paragemmobacter* formed a separate phylogenetic line with *Gemmobacter.* The genomic size was 3.6–4.7 Mbp. The genomic G + C content was 61.7–66.6%. The type species is *Paragemmobacter straminiformis*.

#### Description of *Paragemmobacter straminiformis* comb. nov.

*Paragemmobacter straminiformis* (stra.mi.ni.for’mis. L. neut. n. *stramen*, straw; L. masc./fem. adj. suff. *-formis*, form; N.L. masc. adj. *straminiformis*, resembling straw).

Basonym: *Gemmobacter straminiformis* Kang *et al*. 2017.

The description is as given for *Gemmobacter straminiformis* (Kang et al., 2017).

The type strain is CAM-8^T^ (=KACC 19224^T^ = JCM 31905^T^).

#### Description of *Paragemmobacter ruber* comb. nov.

*Paragemmobacter ruber* (ru’ber. L. masc. adj. *ruber*, red).

Basonym: *Rhodobacter ruber* Chen *et al*. 2021.

The description is as given for *Rhodobacter ruber* (Chen et al., 2021).

The type strain is CCP-1^T^ (=BCRC 81189^T^ = LMG 31335^T^).

#### Description of *Paragemmobacter amnigenus* comb. nov.

*Paragemmobacter amnigenus* (am.ni’ge.nus. N.L. masc. adj. *amnigenus*, born in a stream, intended to mean coming from water).

Basonym: *Rhodobacter amnigenus* Chen *et al*. 2021.

The description is as given for *Rhodobacter amnigenus* (Chen et al., 2021).

The type strain is HSP-20^T^ (=BCRC = BCRC 81193^T^ = LMG 31334^T^).

#### Description of *Paragemmobacter kunshanensis* comb. nov.

*Paragemmobacter kunshanensis* (kun.shan.en’sis. N.L. masc. adj. *kunshanensis*, of or pertaining to Kunshan city, Jiangsu province, China, from where the type strain was isolated).

Basonym: *Rhodobacter kunshanensis* Liu *et al*. 2024.

The description is as given for *Rhodobacter kunshanensis* (Liu et al., 2021).

The type strain is HX-7-19^T^ (=KCTC 72471^T^ = CCTCC AB 2020148^T^).

#### Description of *Paragemmobacter aquarius* comb. nov.

*Paragemmobacter aquarius* (a.qua’rius. L. masc. adj. *aquarius*, of or relating to water).

Basonym: *Gemmobacter aquarius* Baek *et al*. 2020.

The description is as given for *Gemmobacter aquarius* (Baek et al., 2020).

The type strain is HYN0069^T^ = KACC 19488^T^ = NBRC 113115^T^).

#### Description of *Pseudogemmobacter blasticus* comb. nov.

*Pseudogemmobacter blasticus* (blas’ti.cus. Gr. masc. adj. *blastikos*, budding, sprouting; N.L. masc. adj. *blasticus*, budding, apt to bud).

Basonym: *Rhodopseudomonas blastica* Eckersley and Dow 1981.

Homotypic synonym: *Fuscovulum blasticum* (Eckersley and Dow 1981) Suresh *et al*. 2020.

The description is as given for *Fuscovulum blasticum* (Suresh et al., 2019).

The type strain is NCIB 11576^T^ (=ATCC 33485^T^ = NBRC 16437^T^).

#### Description of *Pseudothioclava nitratireducens* comb. nov.

*Pseudothioclava nitratireducens* (ni.tra.ti.re.du’cens. N.L. masc. n. *nitras*, nitrate; L. pres. part. *reducens*, converting to a different state; N.L. part. adj. *nitratireducens*, reducing nitrate).

Basonym: *Defluviimonas nitratireducens* Liu *et al*. 2017.

The description is as given for *Defluviimonas nitratireducens* (Liu et al., 2017b).

The type strain is DL5-4^T^ (=MCCC 1A06955^T^ = LMG 29616^T^).

#### Description of *Paenirhodobacter ferrireducens* comb. nov.

*Paenirhodobacter ferrireducens* (fer.ri.re.du’cens. L. neut. n. *ferrum*, iron; L. pres. part. *reducens*, reducing; N.L. part. adj. *ferrireducens*, reducing iron).

Basonym: *Sinirhodobacter ferrireducens* corrig. Yang *et al*. 2018.

The description is as given for *Sinorhodobacter ferrireducens* (Yang et al., 2013).

The type strain is SgZ-3^T^ (=KACC 16603^T^ = CCTCC AB 2012026^T^).

#### Description of *Paenirhodobacter huangdaonensis* comb. nov.

*Paenirhodobacter huangdaonensis* (huang.dao.nen’sis. N.L. masc. adj. *huangdaonensis*, of Huangdao, a district of Qingdao city in Shandong province, PR China, where the type strain was first isolated).

Basonym: *Sinirhodobacter huangdaonensis* corrig Xi *et al*. 2019.

The description is as given for *Sinorhodobacter huangdaonensis* (Xi et al., 2017).

The type strain is L3^T^ (=CGMCC 1.12963^T^ = KCTC 42823^T^).

#### Description of *Paenirhodobacter populi* comb. nov.

*Paenirhodobacter populi* (po’pu.li. L. gen. n. *populi*, of the poplar tree, of the genus *Populus*).

Basonym: *Sinorhodobacter populi* Xu *et al*. 2019.

The description is as given for *Sinorhodobacter populi* (Xu et al., 2019).

The type strain is sk2b1^T^ (=CFCC 14580^T^ = KCTC 52802^T^).

#### Description of *Paenirhodobacter hankyongi* comb. nov.

*Paenirhodobacter hankyongi* (hank.yong’i. N.L. gen. n. *hankyongi*, of Hankyong National University, where the strain was identified).

Basonym: *Sinirhodobacter hankyongi* Lee *et al*. 2020.

The description is as given for *Sinirhodobacter hankyongi* (Lee et al., 2020).

The type strain is BO-81^T^ (=KACC 19677^T^ = LMG 30808^T^).

## Data availability statement

The data presented in the study are deposited in the online repository, accession numbers OR533672, JAVQHL000000000 and JAVQHM000000000 at https://www.ncbi.nlm.nih.gov.

## Author contributions

ZH: Writing – original draft, Writing – review & editing. ML: Writing – original draft. AO: Writing – review & editing. QL: Writing – review & editing.

## Footnotes

^1^ https://lpsn.dsmz.de/family/paracoccaceae

^2^ https://blast.ncbi.nlm.nih.gov/Blast.cgi

^3^ https://github.com/najoshi/sickle

^4^ https://www.genome.jp/tools/kaas/

^5^ https://ggdc.dsmz.de/

^6^ https://github.com/dparks1134/CompareM

^7^ https://www.ncbi.nlm.nih.gov/genome/browse#!/prokaryotes/Paracoccaceae

^8^ https://lpsn.dsmz.de/genus/paracoccus
