# Supplementary figures and images for: Genome-based analysis of the family Paracoccaceae and description of Ostreiculturibacter nitratireducens gen. nov., sp. nov., isolated from an oyster farm on a tidal flat
Source: Front Microbiol. 2024 Apr 30;15:1376777. doi: 10.3389/fmicb.2024.1376777 (PMC11092380; doi:10.3389/fmicb.2024.1376777)

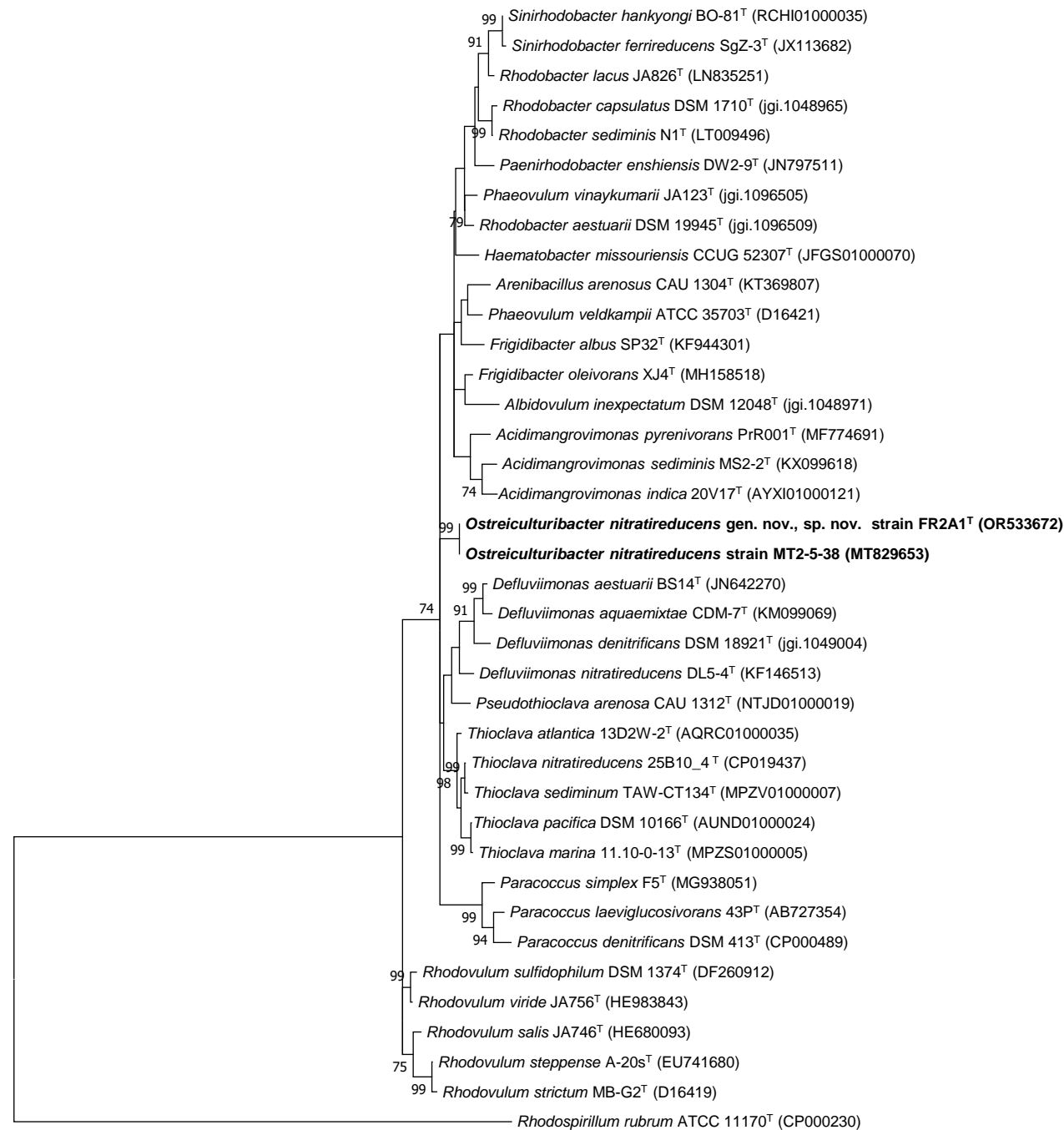

0.1

Supplement: Supplementary file 1 [file Data_Sheet_1.PDF]

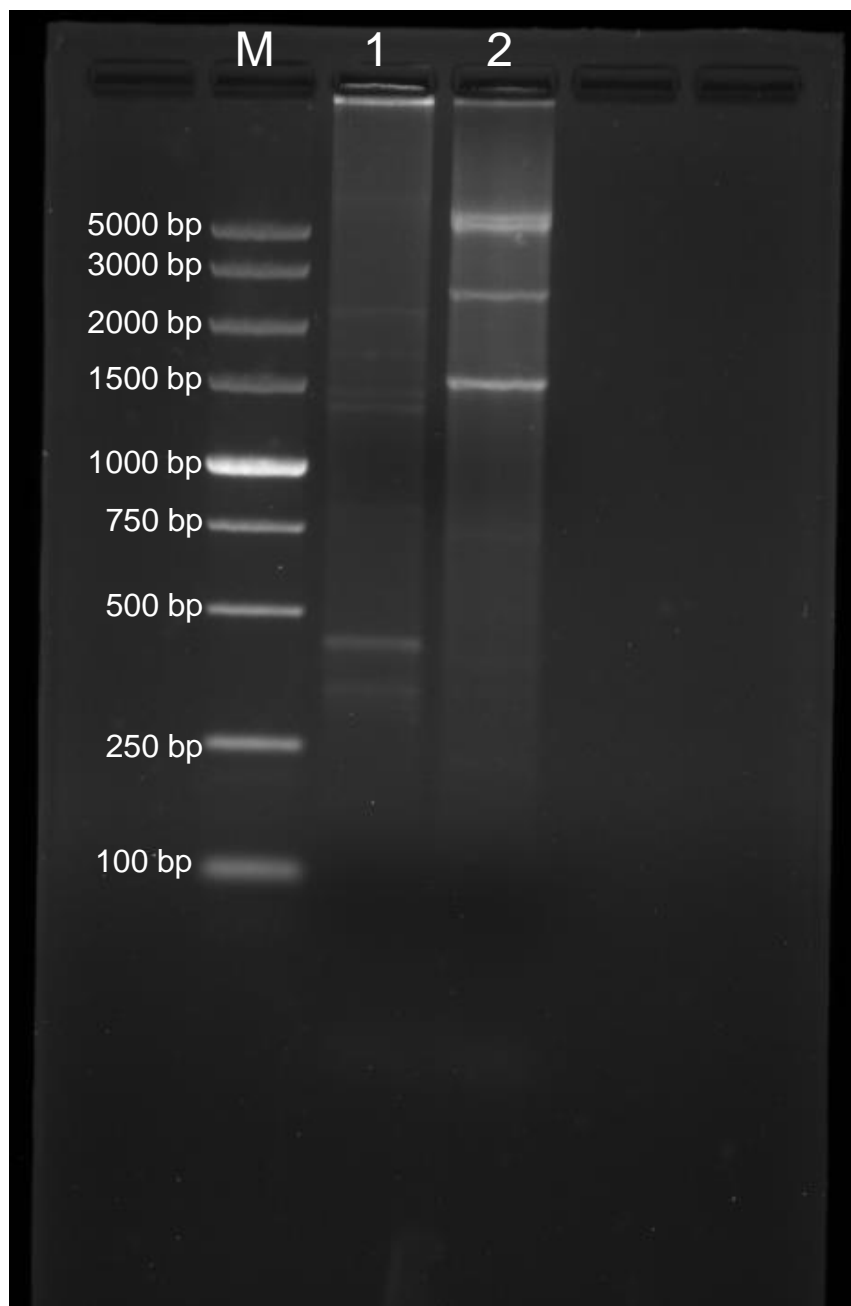

Supplement: Supplementary file 2 [file Data_Sheet_2.PDF]

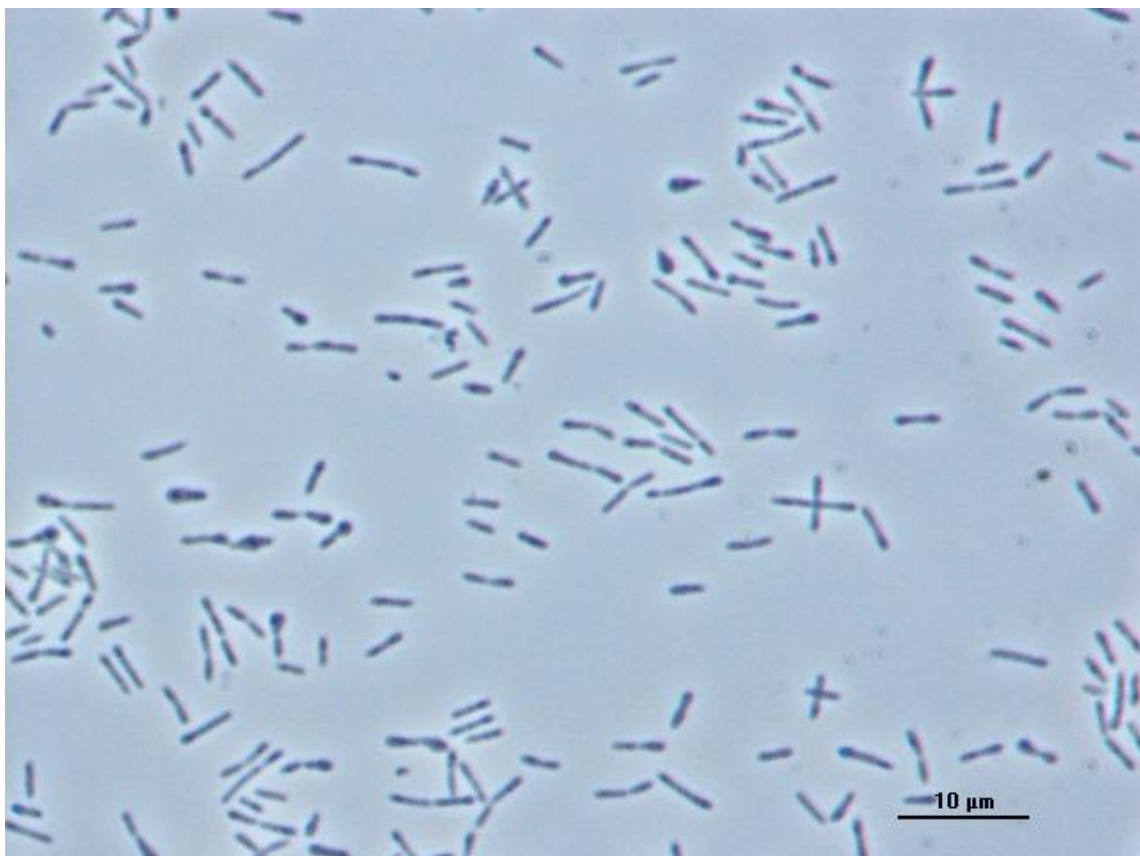

A

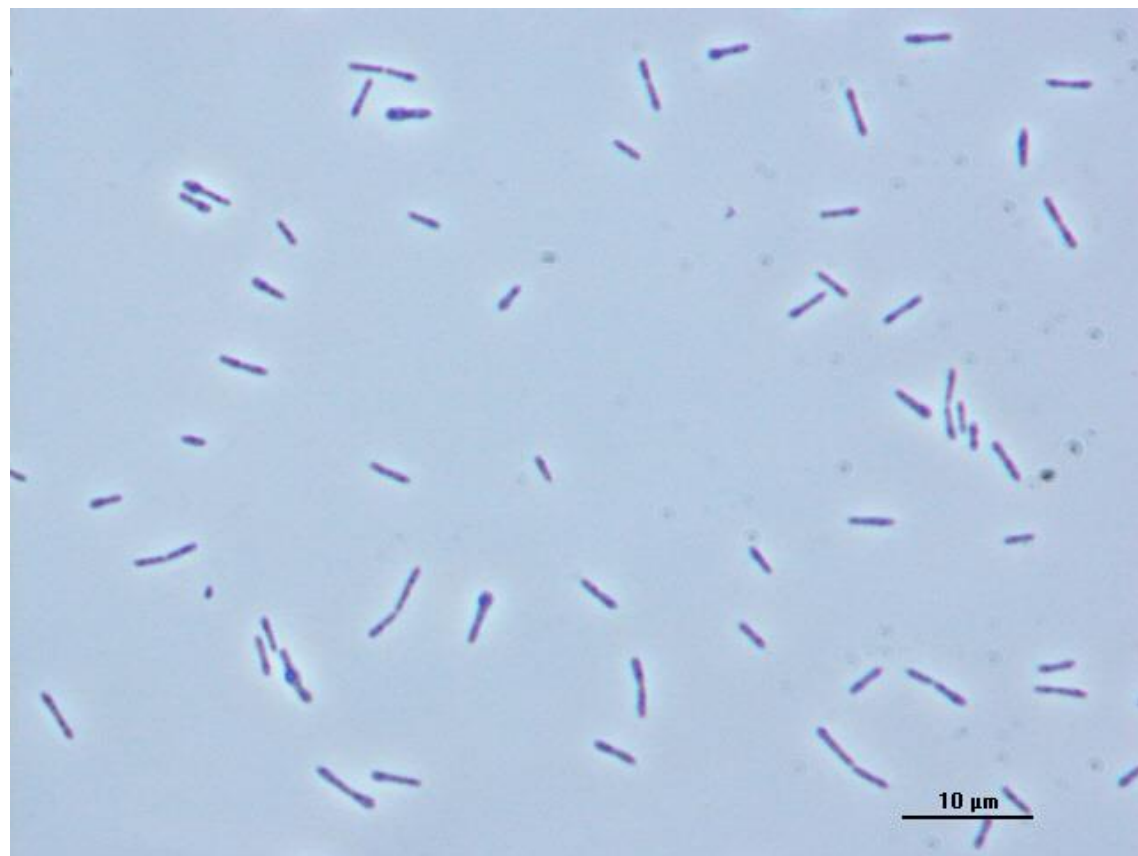

B

Supplement: Supplementary file 3 [file Data_Sheet_3.PDF]

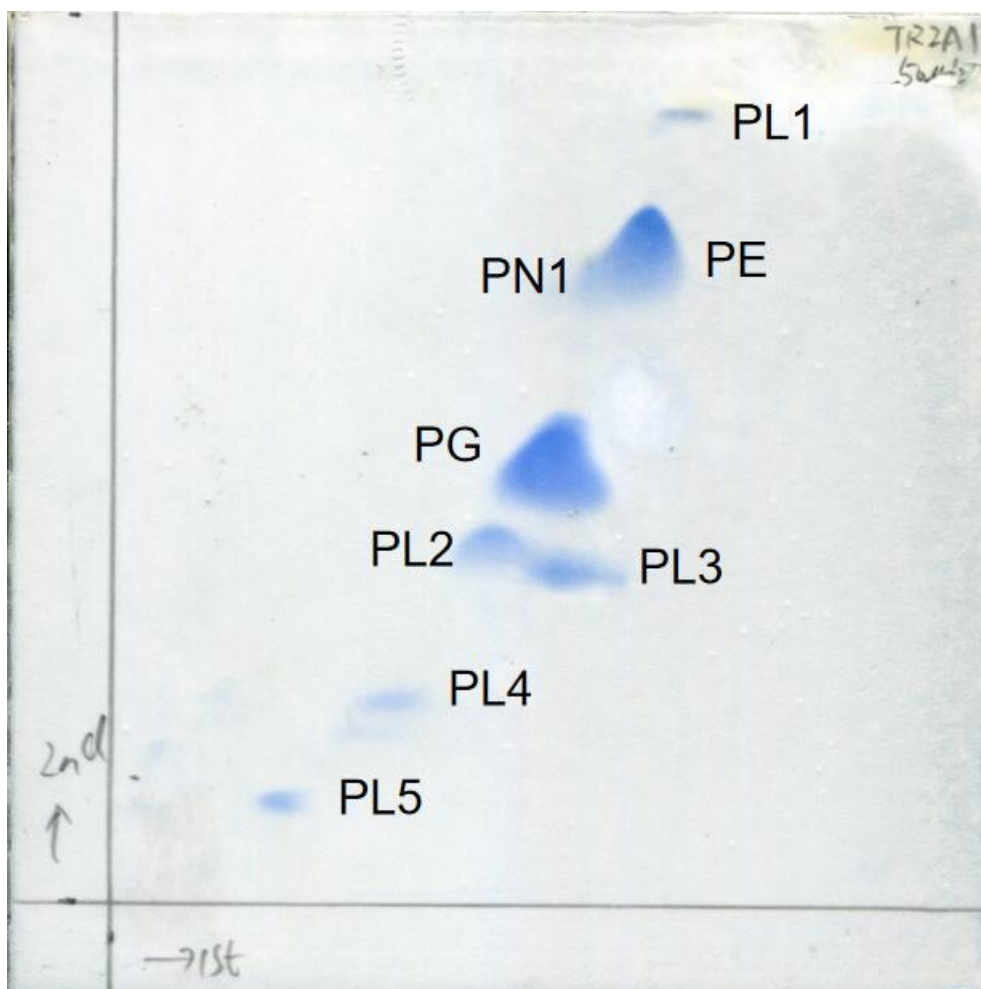

Supplement: Supplementary file 4 [file Data_Sheet_4.PDF]

Tree scale: 0.1

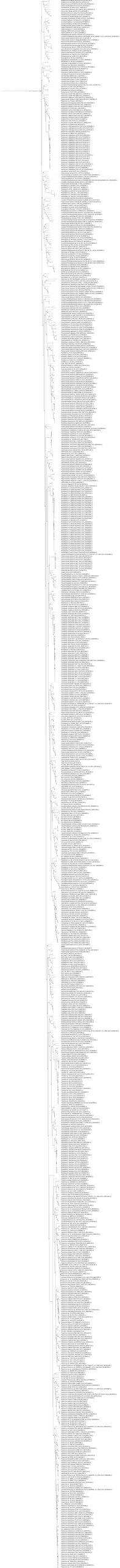

Supplement: Supplementary file 5 [file Data_Sheet_5.PDF]

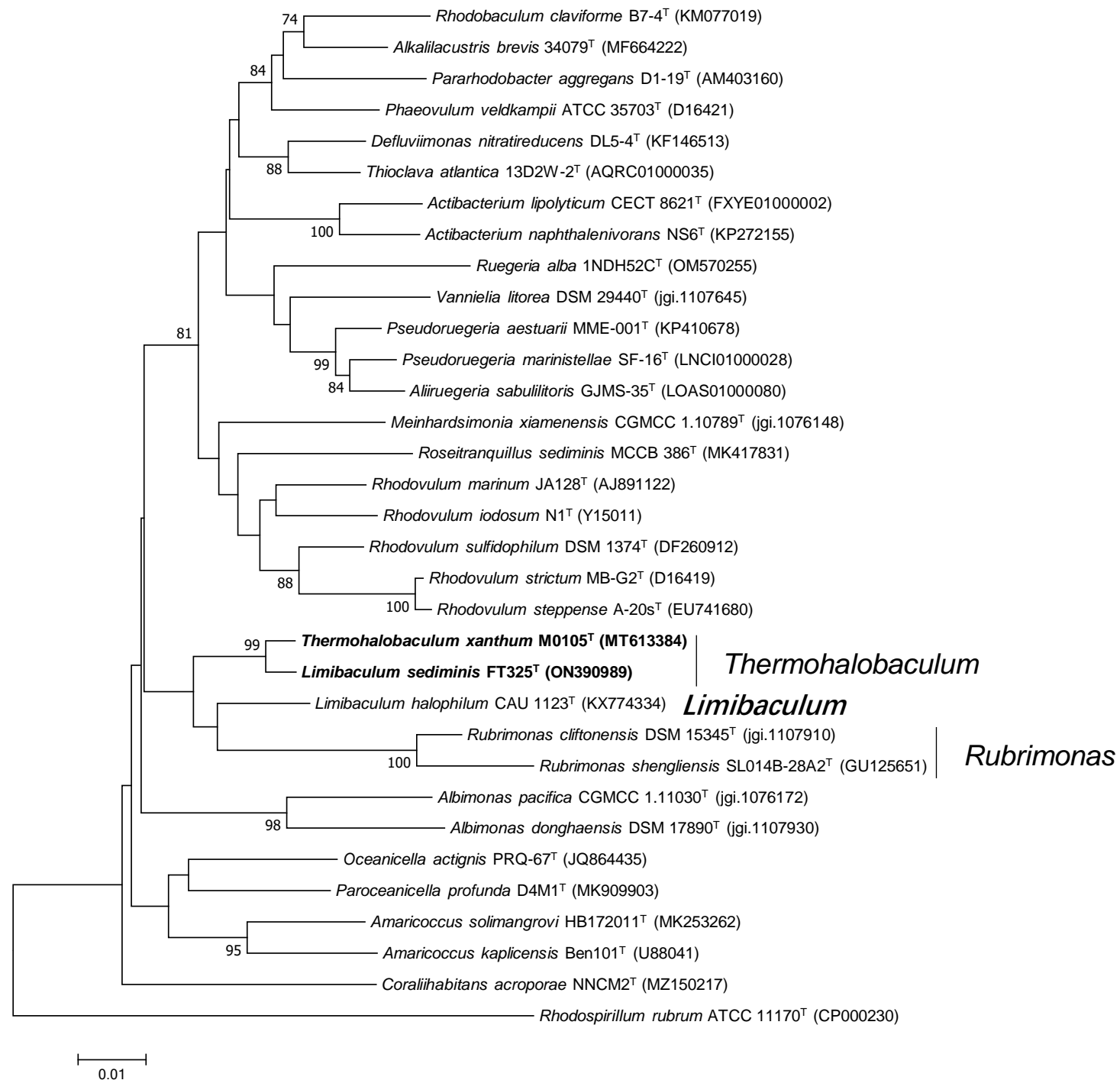

Supplement: Supplementary file 7 [file Data_Sheet_7.PDF]

AAI values

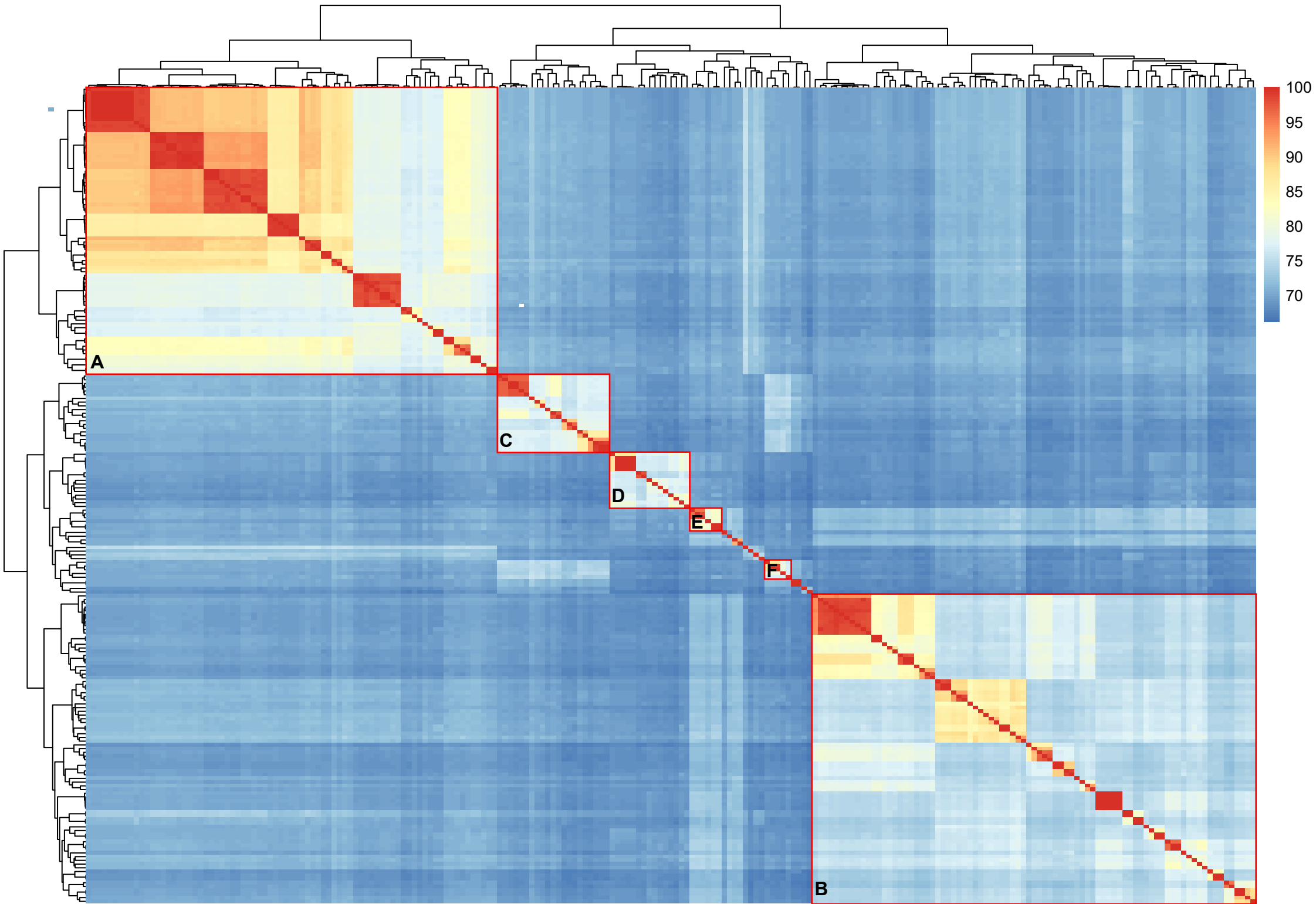

Supplement: Supplementary file 8 [file Data_Sheet_8.PDF]
