# Supplementary material for: Genome-based analysis of the family Paracoccaceae and description of Ostreiculturibacter nitratireducens gen. nov., sp. nov., isolated from an oyster farm on a tidal flat
Source: Front Microbiol. 2024 Apr 30;15:1376777. doi: 10.3389/fmicb.2024.1376777 (PMC11092380; doi:10.3389/fmicb.2024.1376777)
Supplement: Supplementary file 6 [file Data_Sheet_6.PDF]

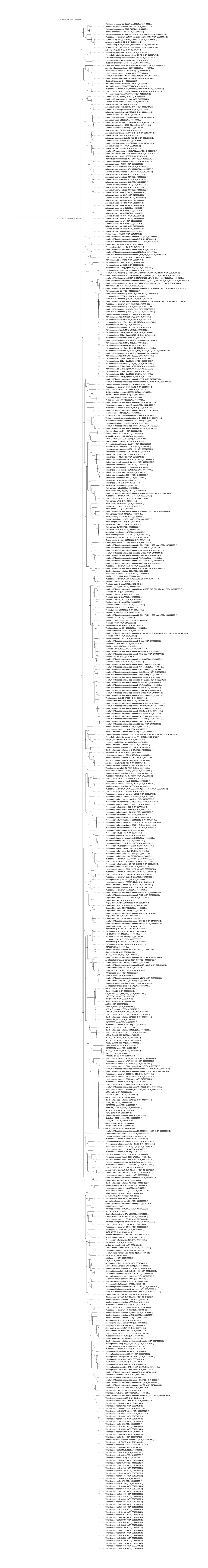
Tree scale: 0.1
